# Biocompatible Nanomaterials as an Emerging Technology in Reproductive Health; a Focus on the Male

**DOI:** 10.3389/fphys.2021.753686

**Published:** 2021-11-11

**Authors:** Barbara Fraser, Alexandra E. Peters, Jessie M. Sutherland, Mingtao Liang, Diane Rebourcet, Brett Nixon, Robert J. Aitken

**Affiliations:** ^1^Priority Research Centre for Reproductive Science, University of Newcastle, Callaghan, NSW, Australia; ^2^Pregnancy and Reproduction Program, Hunter Medical Research Institute, New Lambton Heights, NSW, Australia; ^3^Priority Research Centre for Reproductive Science, School of Biomedical Science and Pharmacy, University of Newcastle, Callaghan, NSW, Australia

**Keywords:** nanomaterials, reproductive research, male reproductive health, chemotherapy, imaging

## Abstract

A growing body of research has confirmed that nanoparticle (NP) systems can enhance delivery of therapeutic and imaging agents as well as prevent potentially damaging systemic exposure to these agents by modifying the kinetics of their release. With a wide choice of NP materials possessing different properties and surface modification options with unique targeting agents, bespoke nanosystems have been developed for applications varying from cancer therapeutics and genetic modification to cell imaging. Although there remain many challenges for the clinical application of nanoparticles, including toxicity within the reproductive system, some of these may be overcome with the recent development of biodegradable nanoparticles that offer increased biocompatibility. In recognition of this potential, this review seeks to present recent NP research with a focus on the exciting possibilities posed by the application of biocompatible nanomaterials within the fields of male reproductive medicine, health, and research.

## Introduction

The last four decades have witnessed the development of a range of nanopharmaceuticals designed for the targeted, efficacious delivery of bioavailable drugs (Masood, 2016; Liu et al., 2018; Liu and Su, 2019; Li et al., 2020). The general strategy has been to encapsulate the payload (reagent, drug combination, or imaging agent) within a dendrimer, liposome, micelle, nanocrystal, or polymer based nanoparticle (NP). An important advantage afforded by nanopharmaceuticals is an attendant increase in the payload therapeutic index, whereby systemic cytotoxicity and off-target effects associated with the free drug are reduced (Bennet and Kim, 2014). Emerging evidence suggests that nanomaterial-based reagents may hold promise in a range of clinical and laboratory-based settings, including those relevant to reproductive health.

The reproductive system functions to arouse sexual interest, produce healthy gametes, facilitate fertilization of the egg, and to provide a nurturing environment for the resultant embryo until the time of parturition. The sophistication and complexity of this system increases its vulnerability to various forms of pathology, which commonly manifest in sub-/in- fertility phenotypes. Indeed, infertility afflicts an estimated 9% of the human population of reproductive age (Punab et al., 2017), representing over 50 million couples globally (Inhorn and Patrizio, 2015). The reproductive system therefore provides many avenues for the therapeutic application of NPs. Indeed, NPs have the potential to be used to target reproductive cells with greater specificity, efficacy and, depending on the cargo, reduced off-target cytotoxicity than conventional reagents. In recognition of this potential, this review considers the advantageous properties, as well as the limitations, of some of the NPs deemed most appropriate for application in the reproductive setting. This review seeks to give an overview of those most commonly used nanoparticles that are biodegradable and biocompatible as well as providing the current state of research in this emerging field.

## Challenges of Targeting the Male Reproductive System

The *in vitro* study of male gametes is complicated by their relative short lifespan *ex vivo* and the fact that they are highly sensitive to environmental exposures as diverse as excursions in temperature and pH as well as to a plethora of xenobiotic compounds (Aitken, 2018; Aitken and Drevet, 2020). *In vivo*, the highly specialized physiology of the mammalian reproductive system, particularly the male reproductive system, provides several unique challenges to the design and implementation of drug delivery strategies. *In vivo* challenges include the particular sensitivity of germ cells and gametes to xenobiotic-induced DNA damage and lipid peroxidation (Aitken, 2020). Additionally, the blood-testis barrier impedes the delivery of therapeutics to the germ cells (Evans and Ganjam, 2011).

### The Fundamental Process of Spermatogenesis

**FIGURE 1:**
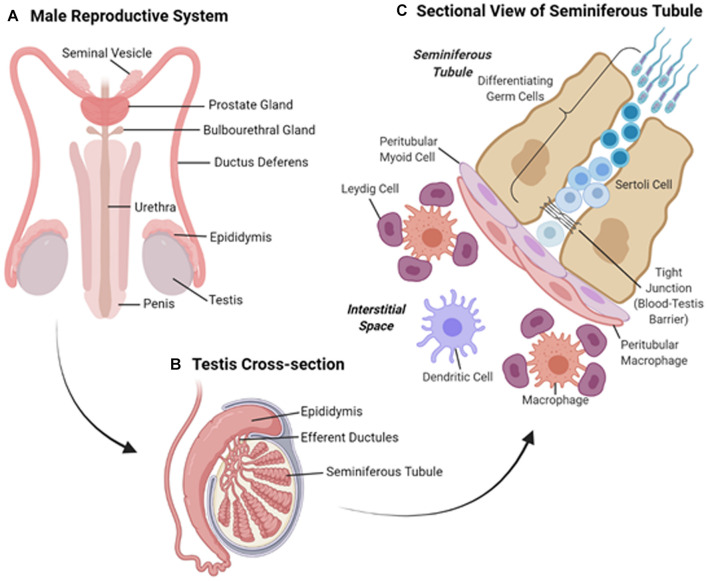
**(A)** Overview of the tissues comprising the human male reproductive system. **(B)** Schematic diagram of a cross-section of the test is showing the coiled seminiferous tubules and adjoining epididymis. **(C)** Sectional view of the cells comprising the seminiferous tubules and surrounding interstitium.

The human male reproductive system (Figure 1A) includes the paired testes (the site of spermatogenesis and steroidogenesis, see below) located in the scrotum and an excurrent duct system, comprising the paired efferent ductules, epididymides, vas deferens and the urethra into which they drain (Figures 1A,B). The urethra runs through the penis through which mature sperm and secretions contributed by the accessory sex glands of the ampullae, seminal vesicles, prostate and bulbourethral glands, travel on ejaculation (Evans and Ganjam, 2011).

The testes (Figure 1B) are broadly divided into two compartments, each of which performs discrete functions; the production of spermatozoa in the seminiferous tubules and the synthesis of steroids in the cells of the surrounding interstitium. The seminiferous tubules support the production of male germ cells through the process of spermatogenesis before discharging morphologically mature sperm cells into the rete testes in preparation for their passage through the excurrent ducts of the male reproductive tract (Hermo et al., 2010). As the vehicle for the transmission of genetic information to the progeny, developing waves of germ cells feature prominently throughout the seminiferous tubules, and are nursed through this process by Sertoli cells (SCs), which constitute the epithelial lining that delineates the seminiferous tubules (Figure 1C). In adulthood, SCs are a mitotically inactive cell population that provides the essential nourishment to guide the survival and morphological remodeling of the developing germline. Additionally, SCs fulfill critical roles in the maintenance of Leydig cells (LC), a highly specialized population of androgen producing cells that reside within the interstitial space, as well as supporting peritubular myoid function in the adult testes and regulating gonadotrophin, particularly follicle stimulating hormone (FSH), output by the brain (Rebourcet et al., 2014).

Along with their support of spermatogenesis, SCs also form networks of tight junctions with adjacent SCs to create a physical barrier known as the blood-testis barrier (BTB), which separates the seminiferous tubules into basal and adluminal compartments. Sertoli cells secrete a number of cytokines, growth factors and immunoregulatory molecules thus protecting the haploid germ cells from the immune system (Stanton, 2016). By establishing a local state of immune tolerance (Chen Q. et al., 2016), the BTB confers immune privilege to the testis. The presence of the BTB has significantly hampered delivery of drugs, such as non-hormonal contraceptive agents, to the adluminal cells (Chen H. et al., 2016). However, certain classes of NP are able to penetrate the BTB and thus hold promise for the delivery of imaging and therapeutic agents to the testes. Indirect evidence that engineered NPs within intracellular vesicles can cross physical barriers, such as the BTB, raises the prospect that endocytotic/exocytotic machinery may contribute to the translocation of larger NPs (Pietroiusti et al., 2013). For instance, 70 nm amorphous silica nanoparticles have been shown to cross the BTB without producing any apparent testicular injury (Morishita et al., 2012). Since the testes are situated in the scrotum, they afford relatively easy access for the direct delivery of imaging agents and therapeutics. This principle may allow magnetic NPs to be directly administered and localized to their site of action via an externally applied magnetic field; a strategy that has been speculated could be used to deliver magnetic mesoporous silica NPs, which have been loaded with F5-peptide (to disrupt the BTB) and adjudin (poorly water soluble contraceptive agent), to the testes (Chen H. et al., 2016).

Along with LC (Figure 1C), the interstitial space between the seminiferous tubules contains macrophages, blood and lymphatic vessels and nerves (Kaur et al., 2013). Leydig cells produce testosterone, which is essential for the maintenance of spermatogenesis, masculinisation and male development. Regulation of testicular function in adults is facilitated via the negative feedback loop of the hypothalamus-pituitary-gonadal (HPG) axis. This axis relies on the pituitary secretion of the gonadotrophins, luteinizing hormone (LH) and FSH, which act on LC and SCs expressing the corresponding receptors (LHr and FSHr, respectively). This, along with the intimate crosstalk between the cells of the testis (Sharpe et al., 1990), means that the targeting of specific cells involved in spermatogenesis in the testis may have wider implications. Effects may include dysregulation of spermatogenic cycles as well as disruption of the regulation of the HPG-axis negative feedback loop and, consequently, the maintenance of masculinisation and male development.

There are a number of cellular insults that are capable of disrupting the redox status and thereby inducing germ cell ablation within the testes. Pro-oxidants and sources of intrinsic and extrinsic oxidative stress, such as autoimmune disease, exposure to radiation (Hanoux et al., 2007), xenobiotics and other cytotoxic agents, are capable of inducing germ cell depletion and hence infertility (Aitken et al., 2003, 2004). The overproduction of ROS overwhelms the body’s antioxidant defenses and results in lipid peroxidation (Sanocka and Kurpisz, 2004; Aitken and Baker, 2006), a loss of DNA integrity (Aitken and Roman, 2008; Aitken and Curry, 2011) and the modification of proteins, including enzymes, that are essential to cell survival (Halliwell, 1991; Aitken and Curry, 2011; O’Flaherty and Matsushita-Fournier, 2017). Through this mechanism, ROS can have a profound impact on the developing gametes, which are exquisitely sensitive to oxidative stress (Hoyer et al., 2001; Hanoux et al., 2007; Devine et al., 2012). The process of ROS production has been well documented for a variety of xenobiotics, such as methoxychlor, polycyclic aromatic hydrocarbons (Saradha and Mathur, 2006), phthalates (Wang et al., 2012) as well as transition metals (Halliwell, 1991), and chemotherapeutic agents (Li et al., 2015). For example, rats exposed to the non-steroidal anti-inflammatory drugs naproxen and meloxicam have been found to exhibit decreased sperm counts and motility as well as significant damage to the seminiferous tubules. These drugs affect the reproductive system by inhibiting prostaglandin synthesis and by producing oxidative stress as a secondary effect (Uzun et al., 2015). By decreasing the effective dose, the use of nanoparticles to deliver chemotherapeutic agents, specifically those that are also targeted to particular cell types, means that the potential risk of exposure to damaging xenobiotics can be decreased.

## Nanotechnology

The field of nanopharmaceuticals encompasses the development and use of nanomaterials for therapeutic, diagnostic, and theragnostic modalities. The physicochemical properties of nanomaterials overcome some of the challenges and limitations posed by more traditional therapeutic agents thus enhancing their utility for drug delivery. Such properties include the ultra-small size (usually below 200 nm in diameter Bawarski et al., 2008) and large surface area to mass ratio of NPs (Zhang et al., 2008). Drug delivery via NPs can also improve the therapeutic value of various water-soluble and insoluble therapeutic drugs. Additionally, encapsulation of normally insoluble, lipophilic drugs means that the use of toxic organic solvents can be avoided (Bawarski et al., 2008). Nanoparticles encapsulate and protect the drug from the biological environment, wherein the active component may be subjected to dilution, catabolism, excretion, denaturation or modification (Bennet and Kim, 2014). As an added benefit, NPs can improve absorption into selected tissues (Peer et al., 2007) and enhancement of intracellular penetration (Peer et al., 2007; Kumari et al., 2010). Nanoparticles can be tailored to suit the application in mind and are commonly based on metalloid, metallic and/or organic components. With considerations in design to include high specificity, cellular uptake and intracellular release, as well as ensuring the NP is configured to display biocompatibility and biodegradability and is cost-effective.

**FIGURE 2:**
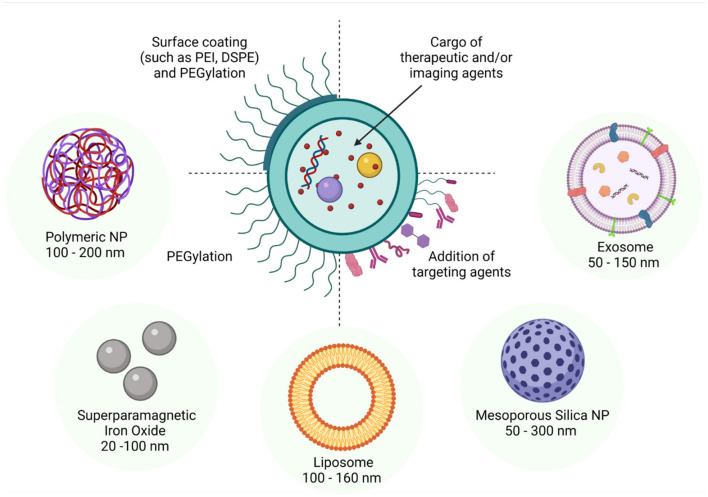
Schematic diagram of some of the more commonly studied nanoparticles highlighting their adaptability for drug encapsulation and surface modification. In line with a spectrum of different applications, such nanoparticles have been formulated to encapsulate cargo as diverse as proteins, peptides, drugs and nucleotides. Similarly, tailored surface coatings can include molecules such as PEG, which confers improved *in vivo* stability, as well as targeting agents such as antibodies, peptides, proteins, and aptamers. The dimensions of the nanoparticles can also be varied through the application of different preparation techniques and surface modifications; with the size range of 90 nm - 120 nm being optimal for facilitating particle uptake via cellular endocytotic pathways. Alternatively, small lipophilic particles can enter the target cell via direct penetration of the membrane while those particles larger than 500 nm generally gain entry via phagocytosis.

There has been an exponential increase in the research and development of nanotechnology over the last several years and, as a reflection, there are currently numerous journals devoted to this research field. Therefore, no single review can cover the entire range of NPs currently being investigated. Fortunately, many thorough reviews detailing the manufacture, properties, applications, advantages, pharmacokinetics, and biosafety of individual categories of NPs have already been published (Zhang et al., 2014; Baek et al., 2015; Sercombe et al., 2015; Wolfram et al., 2015; Ramot et al., 2016; Crucho and Barros, 2017; Manzano and Vallet-Regí, 2019; Sabio et al., 2019; Mitchell et al., 2021). Thus, we have elected to contextualize the implementation of NPs in diagnostic, therapeutic and research applications, with reference to the male reproductive system. In the following section, we provide a brief overview of the major categories of NPs with an emphasis on those spherical NPs within a size range of 10–200 nm and that are thought to be most suitable for *in vitro* and *in vivo* applications in reproductive biology since they are biodegradable, exhibit low bioaccumulation, possess favorable renal clearance properties, and have low toxicity (summarized in Figure 2).

### Inorganic Nanoparticles

#### Metallic Nanoparticles

Metallic NPs have specific properties including magnetic (Fe, Ni, Co) (Anderson et al., 2019; Stueber et al., 2021), antimicrobial (Ag) (Liao et al., 2019) and photothermal (Au) (Sasidharan and Monteiro-Riviere, 2015) that have been harnessed for biomedical applications. However, toxicity (Balasubramanian et al., 2010; Meng et al., 2018), particularly reproductive toxicity (Bawarski et al., 2008; Brohi et al., 2017; Ema et al., 2017; Habas et al., 2021; Vassal et al., 2021), and lack of biocompatibility and biodegradability of metallic NPs means that most of this group of nanoparticles fall outside the scope of this review. However, two groups of metallic nanoparticles that hold considerable potential for imaging applications are quantum dots and iron oxide nanoparticles, which also has a wide diversity of applications.

Quantum dots (QDs) are semiconductor nanocrystals of ultra- small hydrodynamic radius of <10 nm and are utilised for imaging applications rather than drug delivery. QDs possess very desirable qualities such as high yield and stable fluorescence, as well as being amenable to surface modification with targeting moieties. These attributes could make them invaluable in research (Zhou et al., 2007) as well as diagnostic imaging. However, unfortunately, metallic QDs are often composed of heavy metals such as cadmium, lead and arsenic which may leak from the QDs with toxic consequences such as oxidative stress as well as morphological changes and dysfunction of the mitochondria (Liu and Tang, 2020; Nguyen et al., 2020).

Arguably, the most promising of the biodegradable, metal based, nanoparticles are the iron oxide NPs.Iron oxide NPs (IONPs), which have been widely researched for drug delivery and as platforms for magnetic resonance imaging (MRI) contrast reagents. IONPs are biodegraded in vivo into iron ions (Ehlerding et al., 2016), which in the context of a single dose, are believed to exert negligible to no detrimental effects. The extent of biodegradation and accumulation, however, depends on the surface coating applied to the NP. For instance, plasma stability of IONPs was notably improved by the application of polyethylene glycol (PEG)- and heparin- coatings, while PEGylation also substantially decreased plasma clearance due to reduced distribution to the liver and spleen (Lin et al., 2015). The nature of the surface coating has also been found to influence the cellular uptake of the IONPs with chemicals such as polyethylenimine (PEI) and maghemite-rhodium citrate (MRC) both enhancing uptake (Alalaiwe, 2019). To date, and perhaps owing to the possibility of adverse effects from bioaccumulation, these particles have only seen application in clinical imaging procedures as opposed to drug delivery applications. The superparamagnetic properties of SPIONs mean that they are often utilised as MRI contrast reagents. Accordingly, of the 14 current clinical trials using iron-oxide based NPs, almost all are centered on the efficacy of delivering MRI contrast reagents. This same property also makes SPIONs useful for magnetic fluid hyperthermia treatment of cancers where SPIONs are applied either systemically or locally. Under the influence of a sinusoidally alternating magnetic field (AMF), the NPs generate heat, through a process called induction, to above 42°C. The increase of temperature above physiological levels results in apoptosis and cell ablation within the targeted tissue (Dadfar et al., 2020) as well as increasing tumour susceptibility to chemotherapy and radiation (Chandrasekharan et al., 2020).

#### Silica Nanoparticles

Silica nanoparticles (SNPs) are composed of porous silica (Liu et al., 2013) and can have a range of pore sizes, Figure 2. However, mesoporous SNPs (MSNPs) have been most widely studied because of their uniform pore size, which can range from 2 to 50 nm, and long-range ordered pore structure (Jafari et al., 2019). Mesoporous SNPs have been found to provide tunable porosity and size, large surface area, structural diversity, and easily modifiable chemistry for functionalization and increased biocompatibility (Liu et al., 2018).

Over the last 10 years, there have been a plethora of studies exploring the use of MSNPs as delivery agents, particularly for cancer chemotherapeutics see summaries in recent reviews by Croissant et al. (2018), Jafari et al. (2019), Sabio et al. (2019). Mesoporous SNPs have also been the focus of research for bio-imaging and biosensing strategies (Jafari et al., 2019). Although silica NPs have been found to have some cytotoxicity, which is size- and surface charge- dependent, MSNPs exhibit minimal toxicity at less than 2 mM (Sabio et al., 2019) and degrade *in vivo* to silicic acid, which is then excreted via the kidneys (Ruoslahti, 2012). Accordingly, silica-based nanostructures are considered to be safe (Jafari et al., 2019) and ideal (Cha et al., 2017) for biomedical applications. Additionally, haemolysis and toxicity of silica NPs can be considerably reduced by surface modification, particularly via PEGylation (Jafari et al., 2019).

Silica NPs have many advantages, including being inexpensive to prepare as they can be synthesized from commercially available, cost-effective materials (Croissant et al., 2018). MSNPs are very robust and persist in the cell when compared to the more organic based NPs. This property makes them ideally suited for carrying imaging agents for cellular labeling (Huang et al., 2005). Most relevantly for this review, Morishita et al. (2012) found that 70 nm MSNPs were able to penetrate the blood-testis barrier (BTB) and were found in Sertoli and spermatogonial cells 24 h after administration with no adverse effect on the testes. An additional advantage of MSNPs over other nanosystems is that, because of their porous nature, they have a much larger carrying capacity and, depending on the pore size, can shelter large cargo molecules such as proteins or DNA (Croissant et al., 2018). Therefore, MSNPs can deliver more cargo per NP, so minimizing the required dose and the exposure of sensitive reproductive tissue and gametes to possible adverse reactions (Barkalina et al., 2014b).

### Organic Nanoparticles

Synthetic organic nanoparticles and exosomes, which are natural nanoparticles, are discussed below. Polymeric, liposomal and carbon nanoparticles are all examples of synthetic organic nanoparticle. However, we have not included carbon nanoparticles in this overview as carbon nanoparticles, particularly carbon nanotubes, have exhibited bioaccumulation, toxicity and lack biodegradability (Xie et al., 2017; Mohanta et al., 2019; Prajapati et al., 2020).

#### Liposomal Nanoparticles

Liposomal NPs are self-assembling spherical vesicles composed of mixtures of amphiphilic phospholipids and cholesterol arranged in a bilayer surrounding an aqueous core. These vesicles can encapsulate drugs with widely varying lipophilicity as the drug can either be associated with the bilayer or within the entrapped aqueous space. However, liposomes have suffered from one major obstacle for their use as a drug delivery vehicle, that of their rapid removal from circulation, largely through the scavenger function of the RES (Woodle and Lasic, 1992). This sequestration in the RES often leads to accumulation and degradation, along with cellular toxicity and consequent impairment of their activity. Additionally, lipid exchange, particularly with high-density lipoproteins (HDL) in the bloodstream, leads to liposome disintegration (Woodle and Lasic, 1992). Liposomes are limited by this lack of stability, along with poor batch-to-batch reproducibility, difficulties in sterilization and, sometimes, low encapsulation efficiency (Woodle and Lasic, 1992; Bawarski et al., 2008). However, covalent attachment of water-soluble polymers, such as PEG, decreases liposome immunogenicity and reduces uptake by the RES and effectively increases liposome *in vivo* half-life (Allen et al., 1991) from 2 h for non-PEGylated NPs compared to between 15 and 24 h for their PEGylated counterparts (Koo et al., 2005).

Since Gregoriadis and Ryman (1971) first proposed their use as enzymes or drug carriers, research on the formulation of liposomal NPs continues to improve and expand. In fact, a review of current clinical trials, identifies 403 new liposomal nanomedicines in Phase I to Phase IV trials. However, almost all of the newly developed nanomedicines, including those with a liposomal base, have some polymeric component. Amphiphilic block copolymers provide a means to overcome the limitations of liposomes, such as membrane instability and drug retention (Oltra et al., 2014). This then leads us to explore the basis of the next generation of nanomedicines based on formulations of polymeric NPs.

#### Polymeric Nanoparticles

Polymeric nanoparticles (PNPs) constitute a large proportion of the NP formulations that have been approved by the U.S. Federal Drug Administration (FDA) and are currently being marketed (Bobo et al., 2016). Polymeric nanoparticles have been used to encapsulate both hydrophobic and hydrophilic reagents, thus protecting labile therapeutic agents from degradation, and can be used for all parenteral applications (Bawarski et al., 2008). Polymeric nanoparticles have the potential to provide highly specific delivery of the encapsulated drug in a controlled manner as they are tunable for temporal and corporeal control of drug release and can also be engineered to exhibit a finely controlled release rate (Colson and Grinstaff, 2012).

Combined, the stability, responsiveness, tunability, facility for specific functionality via surface modification, along with biocompatibility and FDA approval status afforded to NPs composed of the amphiphilic block copolymers, poly(lactic acid) (PLA) or poly(lactic-co-glycolic acid) (PLGA), has identified this class of NP as the focus for intense scientific and clinical interest (Kumari et al., 2010; Colson and Grinstaff, 2012). As with liposomes and other NPs, PEGylation of the surface of the PNPs can increase their time spent in circulation (Lakkireddy and Bazile, 2016). The very comprehensive recent review on PLGA and PLA based PNPs by Lakkireddy and Bazile (2016) lists over 50 published studies detailing the *in vitro* and *in vivo* performance of PNPs as drug delivery agents. Furthermore, there are currently six polymeric pharmaceutical products in Phase I, II, and III clinical trials (listed as being not recruiting, recruiting and completed).

Even so, whilst efforts to increase circulation time by modifying the surface of NPs has been reasonably successful, a large proportion of NPs are cleared before they reach their target site. As a consequence, recent research has investigated the efficacy of hybridizing NPs with biological components as a means with which to increase the “stealthiness” of NPs *in vivo*. One of these components, the cell-surface “do-not-eat-me” protein, CD47, binds to and signals phagocytes that the cell is “self” thereby inhibiting phagocytosis. Rodriguez et al. (2013) synthesized a 21-amino acid peptide that was based on the binding site of CD47 and, when attached to the surface of NPs, found that this peptide significantly delayed clearance when compared to control NPs formulated with a scrambled peptide. Additionally, Hu and fellow researchers assembled a hybrid NP by harvesting vesicles derived from erythrocyte membranes and extruding these around a PLGA core. In doing so, they found that the erythrocyte-membrane-coated PLGA NPs exhibited significantly retarded *in vivo* clearance compared to that of PEGylated PLGA NPs (Hu et al., 2011). Such findings may indicate that biological hybrid NPs or modified cellular derived vesicles, such as exosomes (described below), may be an attractive alternative to synthetic NPs.

#### Exosomes

Exosomes are nanosized extracellular vesicles (EVs) of endosomal origin formed by the fusion of multivesicular bodies with the plasma membrane (Skotland et al., 2017) and are released by many different cell types in response to both physiological and pathophysiological conditions (van Niel et al., 2018; Zhang et al., 2019). Exosomes can range from 20 nm up to 150 nm, though commonly, research has focussed on those in the range of 30-100 nm (Barile and Vassalli, 2017). Exosomes act as biological information nanocarriers whose cargo of proteins, lipids and nucleic acids, exert an effect on recipient cells to maintain homeostasis as well as having a role in pathology (Kim et al., 2017; Zhang et al., 2019). Exosomes may be either harvested by ultracentrifugation from cells grown in culture, such as pluripotent stem cells, dermal fibroblasts, embryonic stem cells, and mesenchymal stem cells (MSCs) (Das et al., 2019), or directly from the patient’s body fluids, before being suitably modified and returned to the patient.

Exosomes possess a “homing” ability that facilities the targeting of particular recipient cell type(s). Together, their size and endowment with surface “self” proteins enable exosomes to evade recognition and removal by the immune system and bestows excellent biocompatibility (Wu et al., 2016). Exosomes therefore have great potential to be used as extracellular vesicle mimetics for application in theragnostics, drug delivery and vaccines (Kim et al., 2017). They may carry either endogenously produced cargoes, be engineered to produce particular cargoes or be modified after harvest. Endogenous cargoes can be induced by priming cultured cells using specific endogenous factors, signaling factors, heat stress or other environmental or exogenous stressors (Tesfaye et al., 2020), including oxidative stress Das et al. (2019). Post-harvest modification of exosomes might include encapsulation of drugs or nucleic acids as well as chemical modification of the surface by adding, for example, a targeting moiety. In one recent study, exosomal encapsulation of paclitaxel lowered the toxicity and increased the therapeutic index of this chemotherapeutic drug (Pullan et al., 2019). There have been many other studies investigating exosomal delivery of cargo, including small RNAs, proteins, and small chemotherapeutic agents, to various tumor targets (Das et al., 2019).

The current research in this use of biomimicry to produce safer and more effective EV and exosomal nanosystems for drug delivery was comprehensively reviewed by Kim et al. (2017), Lu and Huang (2020). Such consideration has identified a potential limitation in terms of the relatively low loading efficiency of exosomes.

In summary, the major limitations to the wider use of exosomes as carriers include their unfavorable pharmacokinetics (Das et al., 2019), lack of characterisation, and low encapsulation efficiency (Tran et al., 2019). A deeper understanding of the factors controlling the packaging of exosomal contents, along with the mechanisms of release from the source cells and the uptake by target cells, will be required for a wider translation of exosomes to a clinical setting to occur. Nevertheless, clinical trials are ongoing and include studies aimed at treating cancers (Aqil et al., 2019), including melanoma and lung cancer (van Niel et al., 2018). Recently, for example, researchers have investigated a tumor treatment using exosomes that were derived from MSCs to carry mRNA from a prodrug activating gene (Altanerova et al., 2019). Additionally, there are currently ten exosomal-related pharmaceutical products in Phase I, II, and III clinical trials (listed as being not recruiting, recruiting and completed) (ClinicalTrials.Gov, 2020). Importantly in the context of this review, Altanerova et al. (2019) demonstrated that exosomes, which were tagged with iron oxide NPs, crossed the blood-brain barrier (BBB) and were then taken up by glioma cells. A number of other studies have also confirmed this ability of exosomes to cross the BBB (Gilligan and Dwyer, 2017; Liu and Su, 2019). These findings suggests that exosomes may also have the potential to cross the blood-testis barrier and might therefore find application in targeting germ cells that lie within this anatomical barrier.

### Targeting of Nanoparticles

**FIGURE 3:**
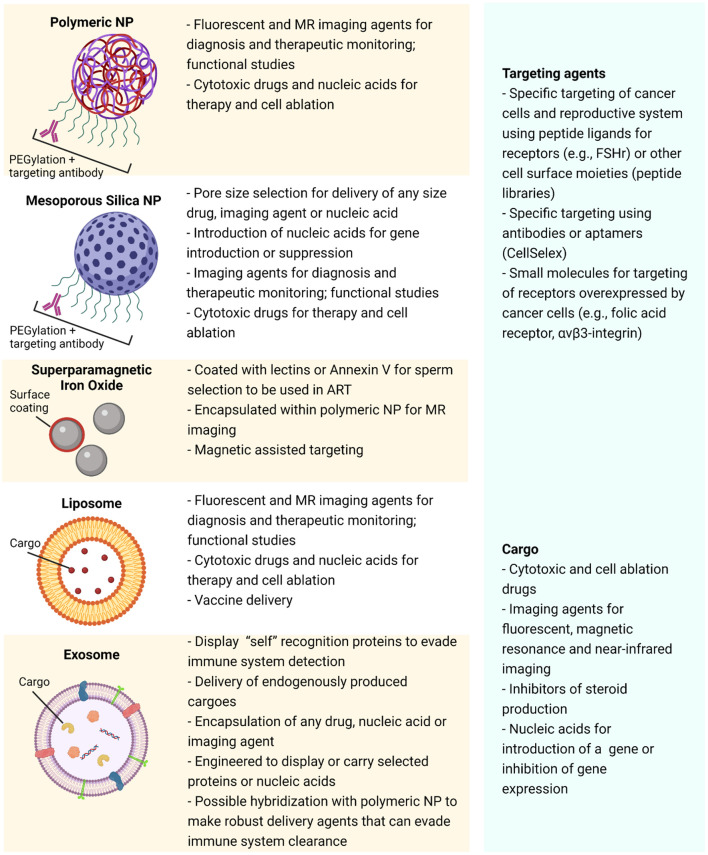
Schematic diagram of some of the more commonly studied nanoparticles highlighting a selection of their properties, possible applications, payloads and targeting agents.

Covalent attachment of targeting moieties such as peptides, antibodies or aptamers to the NP surface provides the opportunity for circulating NPs to bind to specific cell surface biomarkers. This, in turn, facilitates the delivery of the NP payload to selected cell types (Figures 2, 3). Targeting moieties such as peptides can be modeled on agonists or antagonists (Accardo et al., 2014) to well characterized receptors that are unique to the reproductive system or, alternatively, peptides discovered by phage panning of the target cells (Askoxylakis et al., 2011; Ruoslahti, 2012; Le Joncour and Laakkonen, 2018). Aptamers are small oligonucleotide ligands of 15-40 bases that have a 3D structure, which bind to specific targets such as proteins and peptides on the cell surface (Cheng and Liu, 2017). Investigated extensively in cancer therapeutics, antibodies (and monoclonal antibodies in particular), can be used to target the NPs to those cells expressing particular antigens on their outer membrane. Immune cells can also bind to the Fc portion of the intact antibody, thereby initiating a signal cascade that kills the target cell (Peer et al., 2007).

This targeting strategy enables elevation of the local concentration of the drug whilst maintaining the systemic levels well below the maximum tolerated dose (MTD) and thus decreasing the chance of off-target effects. The targeting moieties can be coupled to PEGylated NPs via the PEG portion of the polymer before or after formation of PNPs (Lakkireddy and Bazile, 2016). Targeting components may also be inserted into the outer membrane of the NP. For instance, the use of lipidated moieties, usually with cholesterol-PEG, is a common approach for the bioengineering of exosomes. The hydrophobic cholesterol “tail” embeds in the membrane thereby anchoring the hydrophilic moiety to the membrane surface whilst the targeting hydrophilic end extends outward from the membrane surface (Tran et al., 2019). The targeting moiety must also have a high coupling efficiency and be situated sufficiently distant to the surface of the NP so that it retains its binding conformation and affinity (Accardo et al., 2014). Overall, of the targeting moieties, antibodies are more costly, have increased opsonisation and are more immunogenic than, for example, targeting peptides (Biscaglia et al., 2017).

Advantageously, the testes presents several uniquely expressed receptors for hormones such as the FSH, LH, and the Anti-Mullerian hormone (AMH), that are ideal for targeting using peptides or antibodies. The structure and kinetics of the FSH receptor, in particular, have been extensively studied. On binding of a ligand to its active site, both the FSH receptor and bound ligand are internalized via receptor mediated endocytosis (Shimizu et al., 1987; Shimizu and Kawashima, 1989). Indeed, FSH has already been used as an effective means of targeting contraceptive agents, such as adjudin, to the testes (Mruk et al., 2006).

## Applications of Nanoparticles in Male Reproductive Health, Medicine, and Research

There have been many recent review articles discussing the various aspects of reproductive research, health and medicine that might be addressed using specifically engineered nanosystems (Table 1). Rather than duplicate this effort, our aim is to instead present a selection of the most recent research on NPs that have been applied to the male reproductive system (Supplementary Table 1). Additionally, we highlight the exciting variety of applications where NPs may provide 1. therapeutics for cancer and other diseases with less off-target effects; 2. Improved diagnostics that are minimally-invasive and have increased efficacy; 3. improved gamete quality and genetic manipulation for use in assisted reproductive technologies and 4. by augmenting research capabilities, facilitate greater mechanistic insights into the reproductive process.

**TABLE 1:** Recent review articles exploring nanoparticle use in the male reproductive system.

Review focus	Author and year
Nanomedicine and mammalian sperm- Lessons from the porcine model	Barkalina et al., 2015, 2016
Gene delivery systems for the testes- Gene editing and delivery- male infertility and hypogonadism	Darbey and Smith, 2018
Treatment for male and female infertility using NPs	Abbasi, 2017
Nanotechnology in veterinary medicine -male fertility and sperm function	Falchi et al., 2018
Clinical translation of NPs in human reproduction - Improved therapeutics and early diagnoses reproductive tract conditions	Shandilya et al., 2020
Mesoporous silica NPs for reproductive medicine	Barkalina et al., 2014a
Mesoporous silica NPs and targeted delivery vector for reproductive biology	Barkalina et al., 2014b
Mesoporous silica NPs and their effects on sperm	Barkalina et al., 2014c
NPs in veterinary medicine - Diagnostic, therapeutic and prophylaxis	El-Sayed and Kamel, 2020
Extracellular vesicles used for delivery of molecular compounds to gametes and embryos	Barkalina et al., 2015
Current trends NPs, liposomes, and exosomes for use in semen cryopreservation	Saadeldin et al., 2020
All nanoparticles - Prostate cancer nanodiagnosis and nanotreatment	Barani et al., 2020
Exosomes- Prostate cancer diagnosis, prognosis, therapy	Lorenc et al., 2020
Exosomes - Cancer therapy	Pullan et al., 2019
Exosomes - Stem cell therapy and reproductive medicine	Kharazi and Badalzadeh, 2020
Extracellular vesicles - Urology: fertility, cancer, biomarkers and targeted pharmacotherapy	Tompkins et al., 2015

### Cancers of the Reproductive System

#### Cytotoxic Agents, Targeting, Drug Resistance, and Decreased Sensitivity

Cytotoxic cell ablation pharmaceuticals are currently used for a variety of scientific and medical applications to selectively ablate a single type of cell (Choi et al., 2004) with the main medical application being cancer therapeutics. Nanoparticle technologies meet the criteria of specificity, controllability, and efficacy to a much greater extent than the more traditional, non-targeted, chemotherapeutic approaches that often led to ablation of off-target cells and, consequently, created greater systemic toxicity. Additionally, traditional anti-cancer therapeutics such as doxorubicin, paclitaxel, docetaxel, and captothecin have high lipophilicity and consequent low solubility which combine to limit the deliverable dose (Accardo et al., 2014). Encapsulation in targeted NPs allows effective delivery of these poorly water-soluble drugs, as well as limiting the toxicity of these drugs on non-target tissues (Stylianopoulos and Jain, 2015).

Almost all anticancer therapies rely on the enhanced permeability and retention (EPR) effect, that is, passive accumulation of therapeutics in the tumor (Stylianopoulos and Jain, 2015). The advantages of specific targeting are discussed above and is especially important in the use of the cytotoxic agents for the treatment of cancer so as to minimize off-target effects. Several studies have sought to identify novel targeting molecules for use with cancer therapeutics; with successful examples including acids, peptides, polypeptides, or proteins based on molecules such as the FSH (Zhang et al., 2009, 2013; Fan et al., 2014; Hong et al., 2018), cyclic arginine-glycine-aspartic acid (cRGD) (Zhang Y. et al., 2020), folic acid (FA) (Abou-ElNaga et al., 2017), and hyaluronic acid (HA) (Zhao et al., 2019) that bind to known receptors more abundantly expressed on the surface of reproductive cancer cells. The inclusion of paramagnetic particles (most often superparamagnetic iron oxide NPs) within the nanosystem that may be directed to the target tissue by the application of a magnetic force has been another targeting method exploited by NP researchers (Fathi et al., 2020).

A common problem in cancer therapeutics is the occurrence of resistance to treatment. Strategies for overcoming this phenomenon of drug resistance may include the use of multifunctional NPs containing a combination of the drug and a short interfering RNA (siRNA) payload that inhibits the translation of proteins involved in the resistance pathway. Examples of such dual-mode NPs include those described by Zhang Q. et al. (2020) in the context of ovarian cancer models. Specifically, a polymeric NP containing a photoactivatable Pt(IV)-prodrug and a siRNA directed at the survivin gene (siBIRC5) and targeted using cRGD; a peptide that binds to α_ν_ β_3_ integrins over-expressed on the surface of cancer cells. When exposed to blue-light radiation, this nanosystem was able to increase the accumulation of the drug and to reverse antiapoptotic effects of survivin as well as to enhance ROS levels (Zhang Q. et al., 2020). Another approach to elevate tumor sensitivity may be to utilize different drugs from that applied in the initial treatment regimen. Of course, new drugs require many years of research and development, however, several recent studies have investigated the use of repurposed drugs such as albendazole, a microtubule inhibitor (Noorani et al., 2015) or drugs sourced from plants, such as curcumin (from turmeric), triptolide and celestrol (from *Tripterygium wilfordii* Hook F.) for use in ovarian cancer (Niu et al., 2020). A recent paper by Mohajeri et al. (2020) reviewed the research on the anti-tumor properties of curcumin and, by focussing on curcumin’s purported modulation of oestrogens and androgens, emphasized its use in cancers of the reproductive system. Cancer stem cells (CSC) are thought to be involved in cancer initiation, progression, metastases and recurrence after treatment. Cancer stem cells are also less sensitive to both cytotoxic agents and radiotherapy (Bajaj et al., 2020). Therefore, chemosensitizers and radiosensitizing agents along with specific biomarkers for targeting may be particularly important therapeutically for ablating CSCs (Walcher et al., 2020).

NPs can overcome the challenge of maximizing cytotoxicity within the tumor by incorporating all of the drugs in the one carrier and thereby delivering drug cocktails simultaneously to the cell. The use of nanotherapeutics may also obviate or reduce some of the long- and short-term side effects of chemotherapeutic drugs. For instance, the cardiotoxicity resulting from treatment with cisplatin or doxorubicin. The therapeutic potential of NP cisplatin-delivery strategies have been extensively discussed in review articles by Browning et al. (2017), Cheng and Liu (2017), Deng et al. (2020). Additionally, some researchers have sought to mitigate this toxicity by combining these drugs with a cardioprotective polyphenol such as resveratrol, quercitrin, or curcumin (Al Fatease et al., 2019).

### Cancers of the Male Reproductive System

#### Prostate Cancer

Globally, prostate cancer (PC) is the second most commonly diagnosed cancer in men (Barsouk et al., 2020) and is responsible for 7.1% of all cancer deaths (Bray et al., 2018). The prevalence of PC is around 3-fold in countries with a high income compared to that in low income countries but the mortality rate per 100,000 is around the same (Barsouk et al., 2020). As prostate cancer is diagnosed using serum prostate specific antigen (PSA) levels and biopsy, the higher prevalence data may result from the higher routine screening rates for PSA (Barsouk et al., 2020). Having said this, along with increasing age, the etiology of PC has been linked to a number of genetic and lifestyle factors more commonly observed in high HIC. Genetic markers are the BRCA2 and the HPC (hereditary prostate cancer) genes, while contributory life-style factors include obesity, smoking and a diet high in calcium and dairy products and low in alpha-tocopherol and selenium (Barsouk et al., 2020). First line treatment for PC restricted to the prostate may be radical prostatectomy. However, the prostate gland is difficult to access surgically and prostatectomy may result in unintentional post-surgery consequences such as urinary incontinence and erectile dysfunction (Sebesta and Anderson, 2017). Thus, effective alternative treatments may be preferable, for example, prostate-sparing brachytherapy, where radioactive seeds are implanted within the prostate, may be used with external beam radiotherapy. As prostate cancer is often testosterone-dependent, higher risk patients are also treated with gonadotropin releasing hormone antagonists or agonists. This is called androgen deprivation therapy (ADT) and its purpose is to reduce testosterone levels. Cytotoxic agents, such as docetaxel and cabazitaxel, may also be used in conjunction with ADT or other inhibitors of androgen biosynthesis, such as CYP17a inhibitors (abiraterone is one such inhibitor), for patients with disease progression (Barsouk et al., 2020).

There have been at least two recent reviews regarding NP research specifically intended for use in prostate cancer as detailed in Table 1 (Barani et al., 2020; Imran et al., 2020; Lorenc et al., 2020). The main roles for nanosystems in the treatment of PC may rest with the minimization of off-target effects and increasing the therapeutic index of cytotoxic agents, as well in increasing radiosensitivity by delivery of radiosensitising agents. One nanosystem that has shown promising efficacy against castration resistant PC (CRPC) in Phase I (52 patients) (Von Hoff et al., 2016) and Phase II (42 patients) (Autio et al., 2018) trials is the untargeted BIND-014 PEGylated NP containing docetaxel. BIND-014 was found to be well tolerated and showed promising efficacy for PC patients with chemotherapy-naive metastatic CRPC (Autio et al., 2018). Recently, a number of research strategies have been reported in which traditional cancer agents have been encapsulated together (Li et al., 2019) with agents that enhance the effects of radiotherapy (Liu et al., 2020), with natural compounds (Song et al., 2020) within NPs to produce a synergistic effect in PC therapy, or with imaging agents for detection of tumor tissue (Lian et al., 2017). For instance, curcumin and cabazitaxel have been combined within a lipid-coated polymeric NP targeted using an anti-PSMA (Prostate-Specific Membrane Antigen also known as Glutamate carboxypeptidase II) aptamer (A10-3.2) and applied *in vitro* and in *in vivo* xenograft models of the PSMA-positive/androgen-receptor negative LNCaP cell line and the PSMA-negative/androgen-receptor positive CRPC PC-3 cell line (Chen et al., 2020). *In vitro*, the nanosystem exhibited significantly better cell inhibition than the drug combination in solution. Additionally, after 21 days of treatment of the xenograft nude mice, the aptamer-targeted NPs containing a 2:5 ratio of curcumin to cabazitaxel outperformed both the bare NPs and the unencapsulated drug combination. This nanosystem also exhibited greater *in vivo* half-life, improved targeting, greater accumulation in the tumor and less accumulation in the heart, greater cytotoxicity and significantly reduced tumor volume, as well as improved maintenance of body weight when compared to the free drugs (Chen et al., 2020).

NPs may better facilitate the detection of ultra-small clusters of tumor cells by MRI than traditional contrast reagents. With this in mind, Du et al. (2020) encapsulated the fluorescent Cy7 agent within a PSMA-targeted manganese oxide-MSNP and applied this nanosystem to LNCaP cells in both *in vitro* and *in vivo* settings, to demonstrate higher specific targeting and enhanced near-infrared imaging and MRI compared to that of the untargeted NP or the Cy5/Mn_3_O_4_ alone. However, imaging agents may also be combined with cytotoxic agents within the same NP. One such system was recently reported by Fang et al. (2020) who combined docetaxel and a cluster of superparamagnetic iron nanoparticles (SPIONs) within a PLGA-PEG NP for targeted delivery using a Wy5a aptamer (selected using SELEX to bind to PC-3 cells). This nanosystem exhibited a significantly lower IC_50_ yet elicited a similar effect on cell viability compared to the drug *in vitro.* Moreover, 8 weeks of *in vivo* treatment of the CRPC mouse model was accompanied by a significant decrease in tumor volume compared to that of the untargeted NP and the docetaxel alone without attendant increase in systemic toxicity. The encapsulated SPIONs facilitated increased contrast enhanced MRI capability when compared to the, commercially available, contrast reagent Resovit (Fang et al., 2020). Similarly, the MRI contrast reagent gadolinium along with cabazitaxel were incorporated within bovine serum albumin NPs by Wan et al. (2020) for concurrent treatment and imaging of PC. These researchers applied this untargeted nanosystem to PC-3 cells *in vitro* and then relied on the enhanced permeability and retention effect for preferential uptake by the tumor xenografts *in vivo*. When compared to the drug alone, this nanosystem enhanced cellular uptake *in vitro* and, *in vivo* retention time was increased (with lower clearance rate), distribution to off-target tissues was reduced, and a significant inhibition of growth of xenograft tumors was observed.

#### Testicular Cancer

Despite being relatively uncommon, accounting for only 1% of all cancers, testicular cancer (TC) is the most prevalent solid malignancy in 15–30 year old males. Like PC, TC is more prevalent in developed countries where altered environmental factors and higher exposure to xenobiotics, both pre- and postnatally, are believed to be responsible for its increasing prevalence (Cheng et al., 2018; Chieffi, 2019). TC is usually detected by the presence of a mass in the scrotum along with scrotal ultrasonography and raised levels of the serum tumor biomarkers α-fetoprotein, β-human chorionic gonadotrophin, lactate dehydrogenase, and miRNA 371a-3p (Ghoreifi and Djaladat, 2019). Currently, the overall 5-year survival rate for testicular tumors is 95% (Ghoreifi and Djaladat, 2019).

Treatment of TC usually involves removal of the effected testis (orchiectomy) or chemotherapy followed by orchiectomy in the case of metastasis (Ghoreifi and Djaladat, 2019). Testis sparing surgery is used where possible to maintain testosterone levels and to preserve fertility (Ghoreifi and Djaladat, 2019). Chemotherapy usually comprises four cycles of bleomycin, etoposide, and cisplatin (BEP) or four cycles of etoposide, ifosfamide, and cisplatin (VeIP) if bleomycin is contraindicated (Chen and Daneshmand, 2018). However, systemic administration of cisplatin, considered essential for testicular cancer treatment, comes with the cost of possible nephrotoxicity, ototoxicity and neurotoxicity (Browning et al., 2017) along with longer-term cardiovascular problems and metabolic syndrome associated with lowered testosterone levels (Poniatowska et al., 2020).

Studies of nanosystems specifically aimed at TC are rare – perhaps due to its relatively low incidence – however, one such study by Al-Musawi et al. (2021), covalently attached the anticancer agent vincristine to a SPION and encased this entity in dextran with surface folic acid to develop a targeted and directable NP. These NPs demonstrated a 10-fold increase in toxicity compared to the free drug when applied to Tera-1 testicular tumor cells *in vitro* (Al-Musawi et al., 2021). Attention has also increasingly focused on the longer-term treatment sequelae as up to 60% of survivors of TC face the aforementioned consequences of lowered testosterone levels corresponding to the cumulative dose of cisplatin (Poniatowska et al., 2020). Encapsulation of cisplatin and other more toxic chemotherapy agents in NPs may provide a way of protecting cancer patients from associated long-term side-effects by improving the therapeutic index through specific targeting and controlled release.

### Reproductive Health and Fertility

#### Infertility

As well as facilitating targeted gene delivery, nanosystems may prove to be powerful research tools in the investigation of the causes of infertility as well as providing platforms for diagnostics and therapeutics. Several recent studies have demonstrated the safety and efficacy of MSNPs for this application, particularly for nucleic acid delivery. In this context, Barkalina et al. (2014c) demonstrated that incubation of spermatozoa with MSNPs had minimal impact on DNA integrity, motility, viability, or acrosome morphology. Additionally, PLGA-PEG polymeric NPs encapsulating fluorescein isothiocyanate (FITC) were shown to be taken up by cumulus-enclosed oocytes in a proof-of-concept study (Goncalves et al., 2020). These findings, along with the fact that MSNPs have been shown to be ideal carriers of genetic material (i.e., siRNA and miRNA) (Pecot et al., 2011; Cha et al., 2017), raises the prospect that NPs may be an ideal alternative to electroporation or the use of viral vectors for the transfer of genetic material to gametes. Current non-viral, viral and liposomal delivery systems that have been used for gene editing in the testes were explored in a recent review by Darbey and Smith (2018). Gene editing using the CRISPR-Cas9 technology has many potential uses in pluripotent stem cells, gametes and the embryo. It may be particularly useful for male infertility where it could be used to edit spermatogonial stem cells, especially for individuals afflicted with spermatogenic dysfunction and an attendant inability to produce mature spermatozoa (Niederberger et al., 2018). Controlled testicular release of a missing/aberrantly expressed protein could conceivably restore fertility (albeit temporarily). For example, intratesticular delivery of cationic lipid-coated fibroin NPs encapsulating, and slowly releasing, peptidylprolyl isomerase I (PIN1) protein to *Pin1*-knockout mice restored spermatogonial proliferation and BTB integrity (Kim et al., 2020).

Preserving fertility during chemotherapy or restoring fertility after treatment with genotoxic agents or after inflammatory pathology has been the focus of much NP research. Restoring male fertility by cryopreservation of prepubertal testicular tissue (Vermeulen et al., 2017) and post-tumor *in vitro* removal of tumor cells from spermatogonial cells for transplantation in testis after cancer treatment (Shabani et al., 2018) have both met with some success. Amelioration of negative impacts of Ritalin (methylphenidate), commonly used for attention deficit hyperactivity disorder, on fertility by concurrent delivery of SPIONs carrying curcumin was assessed by Raoofi et al. (2020). Curcumin SPIONs were administered concurrently with methylphenidate to rats. Testes weights were maintained whilst enhanced levels of testosterone and improved sperm and serological parameters were observed along with downregulated proinflammatory and proapoptotic genes.

#### Assisted Reproduction Technologies

In the fertilization process, the ejaculated sperm undergo selection as they travel through the female genital tract. This process physiologically modifies the sperm and selects for motile spermatozoa that are capable of undergoing the acrosome reaction, thus ensuring that only the best sperm reach the oocyte for fertilization (Raimondo et al., 2020). When natural conception is not possible, fertilization can be assisted either *in vivo* by artificial insemination or in vitro before implantation of the early stage embryo *in vivo*. Since the birth of the first *in vitro* fertilization (IVF) baby in 1978, the field of assisted reproduction technology (ART) has made much progress. However, among the many challenges that remain in this field, at least some may be addressed using nanotechnology. Thus, non-human models have been used to test the efficacy and safety of nanotechnology in improving ART outcomes. The selection of good quality, healthy spermatozoa for use in artificial insemination (AI), IVF, and intracytoplasmic sperm injection (ICSI) may improve the health of the embryos, the chances of conception, and the viability of the resulting pregnancies. After undergoing a selection process, the sperm sample should show improved motility, and morphology and should exhibit lower levels of DNA fragmentation, DNA oxidative damage and contamination with apoptotic sperm or other cell types (Marzano et al., 2020). The use of NPs in semen cryopreservation (Saadeldin et al., 2020), healthy sperm selection, and removal of damaged spermatozoa from semen samples are promising approaches to improving the quality of sperm samples. Magnetic assisted cell sorting (MACS) utilises surface-modified magnetic NPs included in a column inside an external magnet (Marzano et al., 2020). Damaged and apoptotic sperm remain attached to the magnetic beads whilst the flow through carries the better quality sperm from the column. Nanoparticles such as magnetic IONPs coated with lectins or a combination of lectins and Annexin V that bind to, and facilitate removal of, damaged spermatozoa have been applied to porcine (Feugang et al., 2015a; Durfey et al., 2019; Feugang et al., 2019) and camel (Rateb, 2020) semen samples prior to downstream application in AI, and to human spermatozoa for subsequent use in ICSI (Pacheco et al., 2020). Additionally, in a study aimed at improving the quality of spermatozoa in cattle semen samples, Farini et al. (2016) used Cell-SELEX to discover ssDNA aptamers that bind to damaged spermatozoa and applied these to the surface of magnetic IONPs. MACS has also been shown to be an effective method for the isolation of viable human sperm (Pacheco et al., 2020) with normal morphology and intact mitochondrial membrane potential (Marzano et al., 2020). Sperm samples processed using MACS followed by density gradient centrifugation (DGC) exhibited a significantly higher percentage of sperm with progressive motility and less sperm with fragmented DNA (Berteli et al., 2017). Indeed, MACS followed by DGC removed over 71% sperm carrying fragmented DNA (Zhang et al., 2018). Additionally, when applied to ICSI, sperm selected by MACS followed by DGC produced a higher proportion of high-quality embryos, pregnancy and implantation rates compared to DGC selected sperm (Ziarati et al., 2017) and lower rates of miscarriage (Sanchez-Martin et al., 2017).

Extracellular vesicles are found in the female reproductive tract of both humans and domestic animals where they are postulated to play roles in follicular development, oocyte maturation, embryo development (O’Neil et al., 2020) and the establishment of pregnancy (Jamaludin et al., 2019; O’Neil et al., 2020). Epididymal EVs, or epididymosomes, are believed to play a major role in the delivery of proteins (Sullivan et al., 2007; Longcore et al., 2009), lipids, and small non-coding RNAs (sncRNAs) (Trigg et al., 2019) to the transiting spermatozoa essential for their maturation as well as conferring protection against redox active species (ROS) released by dying cells (Sullivan, 2015). EVs, termed prostasomes, present in seminal plasma (SP) fuse with the post-ejaculatory spermatozoa to enhance motility (Hoog and Lotvall, 2015). Prostasomes also aide semen liquefaction, prevent microbial infections, facilitate blood coagulation and mediate immunosuppression in the female genital tract (Hoog and Lotvall, 2015). In a study by Rodriguez-Caro et al. (2019), EVs from human seminal fluid were shown to bind endometrial stromal cells (ESCs) *in vitro* and thereafter enhance ESC decidualization, prolactin secretion and ultimately, endometrial receptivity. Moreover, it has been concluded that exosomes contained within SP contribute to the immune functions of SP within the endometrium, particularly in the upper female genital tract (Paktinat et al., 2019). It follows that SP exosomes may find application in preparing the endometrium for embryo transfer during ART cycles (Paktinat et al., 2019). As well as discussing the research that has already been conducted on the use of naturally occurring exosomes on various reproductive pathologies, Kharazi and Badalzadeh (2020) suggest combinations of exosomes for alleviating the symptoms of endometriosis and asthenozoospermia. Certainly, the research to date suggests there is great potential for the use of tailored exosomes for treatment of the reproductive system as well as to enhance the success of ARTs.

#### Fertility Control

Successful reproduction involves multiple hormones and gamete production and maturation as well as fusion of the sperm and oocyte, implantation and embryo development. Cells, receptors and molecules involved in these processes are all possible targets for fertility inhibition. Spermatogonial stem cells and Sertoli cells are terminally differentiated cell types that cannot be replenished by the reproductive system, therefore, these cells in particular, represent ideal targets for fertility control (Aitken and Curry, 2011). Moreover, since the FSH receptors on Sertoli and granulosa cells and LH receptors on Leydig and theca cells are uniquely expressed in the testes and ovaries, these cells can be specifically targeted through ligands that bind to these receptors without creating any off-target effects. Nevertheless, the two most recent studies, conducted by the Yata (Yostawonkul et al., 2017; Pagseesing et al., 2018) laboratory, aimed at creating nanosystems for non-surgical sterilization, albeit *in vitro* only, used NPs that were not targeted. In their 2017 study, they loaded a natural product, α-mangostin, into liposomes for intratesticular injection. In proof-of-concept studies on GC-1 spermatogonia, the NPs induced apoptosis and exhibited antiproliferative activity. Abnormal anatomy of seminiferous tubules was observed in cat testicular explants (Yostawonkul et al., 2017). Similarly, antiproliferative activity in spermatogonial cells *in vitro* and cell death in cultured rat seminiferous tubules was observed in their study after application of liposomal nanoemulsion NPs encapsulating doxorubicin (Pagseesing et al., 2018).

## Conclusion

There is now mounting evidence that supports the use of nanomaterials over more conventional materials in the sphere of reproductive biology research. Clinical applications of NPs in reproductive health extend to germline protection, so as to preserve fertility by directly ameliorating damage or by the fortification of the intrinsic germline defenses against various forms of exogenous and endogenous insult. Conversely, NPs may be used to deliver cytotoxic agents for the ablation of the male/female germ cells (or their support cells) for the purpose of contraception. Additionally, imaging is an emerging application for NPs, which can be engineered to deliver any cargo including MRI contrast reagents and fluorescent tags. This may be particularly useful for such diverse clinical applications as the assessment of the ovarian reserve for family planning or to image metastatic cell clusters of reproductive origin.

This review has sought to highlight the breadth and variety of applications to which NPs may be applied to expand and enhance existing imaging and treatment options for conditions of the male reproductive system. Besides their use in diagnostics, reproductive cancer, contraception, ART and reproductive health are all fields that stand to benefit from future developments in NP technologies. In guiding these developments, important considerations include the formulation of biocompatible, well characterized materials that are soluble or colloidal under aqueous conditions. Similarly, *in vivo* use of NPs will only be possible if they present with minimal toxicity/immunogenicity. In this regard, minimization of off-target effects and increased safety indices could perhaps be best addressed via functionalization of the NP surface to ensure it is engineered with the highest levels of target cell specificity and/or differential uptake efficiency into the targeted, as opposed to non-targeted cells. As additional criteria for clinical use, the NPs would benefit from an extended half-life *in vivo* as well as a long shelf-life.

## Future Perspectives

The use of exosomes, which are furnished with specific ligands targeting them to particular cell types, could be arguably among the most exciting developments in nanotechnology. Potentially, molecular engineering of the source cell to express a desired cargo or targeting peptides to then be packaged into or expressed on the membrane surface of exosomes may result in ingenious delivery agents that are customized to the patient. Innovative hybridisation of exosomes to create a new breed of “stealthy” NPs, with increased *in vivo* half-life and biocompatibility, presents many possibilities and would have a wide application in tailored cancer therapy, gene manipulation in germ cells, as well as a wide range of reproductive biology research applications.

The other exciting developments lie in the field of imaging. Recently, researchers have sought to synthesize quantum dots based on carbon (Alas et al., 2020; El-Shabasy et al., 2021) or silica (Zhu et al., 2019), utilizing ecofriendly synthesis (Iravani and Varma, 2020), which would offer a lower toxicity alternative to metal based QDs (Liu and Tang, 2020). QDs may be utilized in fluorescent imaging for *in vitro* and *in vivo* research as well as in diagnostic imaging, offering higher yield and *in vivo* stability in comparison to other fluorescent tags. In reproductive biology research, possible applications for ligand-modified QDs may include gamete labelling for *in vivo* and *in vitro* tracking, as envisaged by Feugang et al. (2012, 2015b), labelling and *in vivo* imaging of germ cells within the testes, and particle tracking. Additionally, *in vivo* tumour imaging using targeted QDs may aid in cancer treatments and also detect cancer cells for early diagnosis *in vivo* and in *ex vivo* samples.

In the field of reproductive science, IONPs present a number of applications, including the aforementioned use in MACS-facilitated sperm selection, superparamagnetic IONPs may facilitate cell MR imaging and magnetic fluid hyperthermia for targeted cell ablation. Additionally, safer alternatives to QDs, based on IONPs may be developed. For instance, Vasquez et al. used a composite luciferase magnetic NP to label sperm which could then be detected using bioluminescent and UV-Vis-NIR microscopy (Vasquez et al., 2016). Finally, magnetic NPs may in future be utilised to manufacture microrobots. In this research, magnetic IONPs were electrostatically attached to a cellular template so that the resultant microrobot could then be manipulated using a magnetic field. The researchers envisage that this technology may be used to activate motility in non-motile spermatozoa (Khalil et al., 2020; Magdanz et al., 2020, 2021), and be useful for the guided delivery of exogenous DNA or chemotherapeutics (Ebrahimi et al., 2020).

## Author Contributions

RA conceived the review topic, edited all sections, and proposed modifications to section organization and figure preparation. BF prepared the manuscript, organized all sections, and made revisions as proposed by co-authors. AP prepared all figures. JS contributed early editing of reproductive system section of manuscript. ML edited nanoparticle sections. DR contributed to co-writing the male reproductive system section. BN edited all sections and proposed modifications to section organization and figure preparation. All authors contributed to the article and approved the submitted version.

## Conflict of Interest

The authors declare that the research was conducted in the absence of any commercial or financial relationships that could be construed as a potential conflict of interest.

## Publisher’s Note

All claims expressed in this article are solely those of the authors and do not necessarily represent those of their affiliated organizations, or those of the publisher, the editors and the reviewers. Any product that may be evaluated in this article, or claim that may be made by its manufacturer, is not guaranteed or endorsed by the publisher.

## References

[B1] Abbasi M. (2017). Nanoparticles as a promising innovative treatment towards infertility. *J. Infertil. Reprodu. Biol.* 5 1–4.

[B2] Abou-ElNaga A. Mutawa G. El-Sherbiny I. M. Abd-ElGhaffar H. Allam A. A. Ajarem J. (2017). Novel nano-therapeutic approach actively targets human ovarian cancer stem cells after xenograft into nude mice. *Int. J. Mol. Sci.* 18:813. 10.3390/ijms18040813 28417924 PMC5412397

[B3] Accardo A. Aloj L. Aurilio M. Morelli G. Tesauro D. (2014). Receptor binding peptides for target-selective delivery of nanoparticles encapsulated drugs. *Int. J. Nanomed.* 9 1537–1557. 10.2147/IJN.S53593 24741304 PMC3970945

[B4] Aitken R. J. (2018). Not every sperm is sacred; a perspective on male infertility. *Mol. Hum. Reprod.* 24, 287–298. 10.1093/molehr/gay010 29546342

[B5] Aitken R. J. (2020). Impact of oxidative stress on male and female germ cells: implications for fertility. *Reproduction* 159, R189–R201. 10.1530/REP-19-0452 31846434

[B6] Aitken R. J. Baker M. A. (2006). Oxidative stress, sperm survival and fertility control. *Mol. Cell. Endocrinol.* 250, 66–69. 10.1016/j.mce.2005.12.026 16412557

[B7] Aitken R. J. Baker M. A. Sawyer D. (2003). Oxidative stress in the male germ line and its role in the aetiology of male infertility and genetic disease. *Reprod. Biomed. Online* 7, 65–70. 12930576 10.1016/s1472-6483(10)61730-0

[B8] Aitken R. J. Curry B. J. (2011). Redox regulation of human sperm function: from physiological control of sperm capacitation to the etiology of infertility and DNA damage in the germ line. *Anto. Redox Sign.* 14 367–381. 10.1089/ars.2010.3186 20522002

[B9] Aitken R. J. Drevet J. R. (2020). The importance of oxidative stress in determining the functionality of mammalian spermatozoa: a two-edged sword. *Antioxidants* 9:111. 10.3390/antiox9020111 32012712 PMC7070991

[B10] Aitken R. J. Koopman P. Lewis S. E. M. (2004). Seeds of concern. *Nature* 432, 48–52. 15525979 10.1038/432048a

[B11] Aitken R. J. Roman S. D. (2008). Antioxidant systems and oxidative stress in the testes. *Oxid. Med. Cell. Longev.* 1, 15–24. 19794904 10.4161/oxim.1.1.6843 PMC2715191

[B12] Alas M. O. Alkas F. B. Aktas Sukuroglu A. Genc Alturk R. Battal D. (2020). Fluorescent carbon dots are the new quantum dots: an overview of their potential in emerging technologies and nanosafety. *J. Mater. Sci.* 55, 15074–15105. 10.1007/s10853-020-05054-y

[B13] Al Fatease A. Shah V. Nguyen D. X. Cote B. LeBlanc N. Rao D. A. (2019). Chemosensitization and mitigation of adriamycin-induced cardiotoxicity using combinational polymeric micelles for co-delivery of quercetin/resveratrol and resveratrol/curcumin in ovarian cancer. *Nanomedicine* 19 39–48. 10.1016/j.nano.2019.03.011 31022465

[B14] Alalaiwe A. (2019). The clinical pharmacokinetics impact of medical nanometals on drug delivery system. *Nanomedicine* 17 47–61. 10.1016/j.nano.2019.01.004 30664946

[B15] Allen T. M. Hansen C. Martin F. Redemann C. Yau-Young A. (1991). Liposomes containing synthetic lipid derivatives of poly(ethylene glycol) show prolonged circulation half-lives in vivo. *Biochim. et Biophys. Acta* 1066 29–36. 10.1016/0005-2736(91)90246-5 2065067

[B16] Al-Musawi S. Ibraheem S. Abdul Mahdi S. Albukhaty S. Haider A. J. Kadhim A. A. (2021). Smart nanoformulation based on polymeric magnetic nanoparticles and vincristine drug: a novel therapy for apoptotic gene expression in tumors. *Life* 11:71. 10.3390/life11010071 33478036 PMC7835862

[B17] Altanerova U. Jakubechova J. Benejova K. Priscakova P. Pesta M. Pitule P. (2019). Prodrug suicide gene therapy for cancer targeted intracellular by mesenchymal stem cell exosomes. *Int. J. Cancer* 144 897–908. 10.1002/ijc.31792 30098225

[B18] Anderson S. D. Gwenin V. V. Gwenin C. D. (2019). Magnetic functionalized nanoparticles for biomedical, drug delivery and imaging applications. *Nanoscale Res. Lett.* 14:188. 10.1186/s11671-019-3019-6 31147786 PMC6542970

[B19] Aqil F. Munagala R. Jeyabalan J. Agrawal A. K. Kyakulaga A. H. Wilcher S. A. (2019). Milk exosomesnatural nanoparticles for siRNA delivery. *Cancer Lett.* 449 186–195. 10.1016/j.canlet.2019.02.011 30771430

[B20] Askoxylakis V. Zitzmann-Kolbe S. Zoller F. Altmann A. Markert A. Rana S. (2011). Challenges in optimizing a prostate carcinoma binding peptide, identified through the phage display technology. *Molecules* 16 1559–1578. 10.3390/molecules16021559 21321528 PMC6259618

[B21] Autio K. A. Dreicer R. Anderson J. Garcia J. A. Alva A. Hart L. L. (2018). Safety and efficacy of BIND-014, a docetaxel nanoparticle targeting prostate-specific membrane antigen for patients with metastatic castration-resistant prostate cancer: a phase 2 clinical trial. *JAMA Oncol.* 4 1344–1351. 10.1001/jamaoncol.2018.2168 29978216 PMC6233779

[B22] Baek S. Singh R. K. Khanal D. Patel K. D. Lee E. J. Leong K. W. (2015). Smart multifunctional drug delivery towards anticancer therapy harmonized in mesoporous nanoparticles. *Nanoscale* 7 14191–14216. 10.1039/c5nr02730f 26260245

[B23] Bajaj J. Diaz E. Reya T. (2020). Stem cells in cancer initiation and progression. *J. Cell. Biol.* 219:e201911053. 10.1083/jcb.201911053 31874116 PMC7039188

[B24] Balasubramanian S. K. Jittiwat J. Manikandan J. Ong C. N. Yu L. E. Ong W. Y. (2010). Biodistribution of gold nanoparticles and gene expression changes in the liver and spleen after intravenous administration in rats. *Biomaterials* 31 2034–2042. 10.1016/j.biomaterials.2009.11.079 20044133

[B25] Barani M. Sabir F. Rahdar A. Arshad R. Kyzas G. Z. (2020). Nanotreatment and nanodiagnosis of prostate cancer: recent updates. *Nanomaterials* 10:1696. 10.3390/nano10091696 32872181 PMC7559844

[B26] Barile L. Vassalli G. (2017). Exosomes: therapy delivery tools and biomarkers of diseases. *Pharmacol. Ther.* 174 63–78. 10.1016/j.pharmthera.2017.02.020 28202367

[B27] Barkalina N. Charalambous C. Jones C. Coward K. (2014a). Nanotechnology in reproductive medicine: emerging applications of nanomaterials. *Nanomedicine* 10 921–938. 10.1016/j.nano.2014.01.001 24444494

[B28] Barkalina N. Jones C. Coward K. (2014b). Mesoporous silica nanoparticles: a potential targeted delivery vector for reproductive biology? *Nanomedicine* 9 557–560. 24827836 10.2217/nnm.14.18

[B29] Barkalina N. Jones C. Kashir J. Coote S. Huang X. Morrison R. (2014c). Effects of mesoporous silica nanoparticles upon the function of mammalian sperm in vitro. *Nanomedicine* 10 859–870. 10.1016/j.nano.2013.10.011 24200525

[B30] Barkalina N. Jones C. Coward K. (2016). Nanomedicine and mammalian sperm: lessons from the porcine model. *Theriogenology* 85 74–82. 10.1016/j.theriogenology.2015.05.025 26116055

[B31] Barkalina N. Jones C. Wood M. J. Coward K. (2015). Extracellular vesicle-mediated delivery of molecular compounds into gametes and embryos: learning from nature. *Hum. Reprod. Update* 21 627–639. 10.1093/humupd/dmv027 26071427

[B32] Barsouk A. Padala S. A. Vakiti A. Mohammed A. Saginala K. Thandra K. C. (2020). Epidemiology, staging and management of prostate cancer. *Med. Sci.* 8:28. 10.3390/medsci8030028 32698438 PMC7565452

[B33] Bawarski W. E. Chidlowsky E. Bharali D. J. Mousa S. A. (2008). Emerging nanopharmaceuticals. *Nanomedicine* 4 273–282. 10.1016/j.nano.2008.06.002 18640076

[B34] Bennet D. Kim S. (2014). “Polymer nanoparticles for smart drug delivery,” in *Application of Nanotechnology in Drug Delivery*, ed. Sezer A. D..

[B35] Berteli T. S. Da Broi M. G. Martins W. P. Ferriani R. A. Navarro P. A. (2017). Magnetic-activated cell sorting before density gradient centrifugation improves recovery of high-quality spermatozoa. *Andrology* 5, 776–782. 10.1111/andr.12372 28622434

[B36] Biscaglia F. Rajendran S. Conflitti P. Benna C. Sommaggio R. Litti L. (2017). Enhanced EGFR targeting activity of plasmonic nanostructures with engineered GE11 peptide. *Adv. Healthc Mater* 6:596. 10.1002/adhm.201700596 28945012

[B37] Bobo D. Robinson K. J. Islam J. Thurecht K. J. Corrie S. R. (2016). Nanoparticle-based medicines: a review of FDA-approved materials and clinical trials to date. *Pharm. Res.* 33 2373–2387. 10.1007/s11095-016-1958-5 27299311

[B38] Bray F. Ferlay J. Soerjomataram I. Siegel R. L. Torre L. A. Jemal A. (2018). Global cancer statistics 2018: GLOBOCAN estimates of incidence and mortality worldwide for 36 cancers in 185 countries. *CA Cancer J. Clin.* 68 394–424. 10.3322/caac.21492 30207593

[B39] Brohi R. D. Wang L. Talpur H. S. Wu D. Khan F. A. Bhattarai D. (2017). Toxicity of nanoparticles on the reproductive system in animal models: a review. *Front. Pharmacol.* 8:606. 10.3389/fphar.2017.00606 28928662 PMC5591883

[B40] Browning R. J. Reardon P. J. T. Parhizkar M. Pedley R. B. Edirisinghe M. Knowles J. C. (2017). Drug delivery strategies for platinum-based chemotherapy. *ACS Nano.* 11 8560–8578. 10.1021/acsnano.7b04092 28829568

[B41] Cha W. Fan R. Miao Y. Zhou Y. Qin C. Shan X. (2017). Mesoporous silica nanoparticles as carriers for intracellular delivery of nucleic acids and subsequent therapeutic applications. *Molecules* 22:782. 10.3390/molecules22050782 28492505 PMC6154527

[B42] Chandrasekharan P. Tay Z. W. Hensley D. Zhou X. Y. Fung B. K. Colson C. (2020). Using magnetic particle imaging systems to localize and guide magnetic hyperthermia treatment: tracers, hardware, and future medical applications. *Theranostics* 10, 2965–2981. 10.7150/thno.40858 32194849 PMC7053197

[B43] Chen H. Mruk D. D. Xia W. Bonanomi M. Silvestrini B. Cheng C.-Y. (2016). Effective delivery of male contraceptives behnd the blood-testis barrier (BTB)lesson from adjudin. *Curr. Med. Chem.* 23 701–703. 26758796 10.2174/0929867323666160112122724 PMC4845722

[B44] Chen J. Daneshmand S. (2018). Modern management of testicular cancer. *Cancer Treat. Res.* 175 273–308. 30168127 10.1007/978-3-319-93339-9_13

[B45] Chen Q. Deng T. Han D. (2016). Testicular immunoregulation and spermatogenesis. *Semin. Cell Dev. Biol.* 59 157–165. 10.1016/j.semcdb.2016.01.019 26805443

[B46] Chen Y. Deng Y. Zhu C. Xiang C. (2020). Anti prostate cancer therapy: aptamer-functionalized, curcumin and cabazitaxel co-delivered, tumor targeted lipid-polymer hybrid nanoparticles. *Biomed. Pharmacother.* 127:110181. 10.1016/j.biopha.2020.110181 32416561

[B47] Cheng L. Albers P. Berney D. M. Feldman D. R. Daugaard G. Gilligan T. (2018). Testicular cancer. *Nat. Rev. Dis. Primers* 4:29. 10.1038/s41572-018-0029-0 30291251

[B48] Cheng Q. Liu Y. (2017). Multifunctional platinum-based nanoparticles for biomedical applications. *Wiley Int. Rev. Nano. Nanobiotechnol.* 9:1410. 10.1002/wnan.1410 27094725

[B49] Chieffi P. (2019). An up-date on novel molecular targets in testicular germ cell tumors subtypes. *Intractable Rare Dis. Res.* 8 161–164. 10.5582/irdr.2019.01055 31218171 PMC6557231

[B50] Choi Y. J. Ok D. W. Kwon D. N. Chung J. I. Kim H. C. Yeo S. M. (2004). Murine male germ cell apoptosis induced by busulfan treatment correlates with loss of c-kit-expression in a Fas/FasL- and p53-independent manner. *FEBS Lett.* 575 41–51. 10.1016/j.febslet.2004.08.034 15388331

[B51] ClinicalTrials.Gov, (2020). A Web-Based Resource That Provides Patients, Their Family Members, Health Care Professionals, Researchers, and the Public With Easy Access To Information On Publicly And Privately Supported Clinical Studies On A Wide Range Of Diseases And Conditions. Available online at: https://clinicaltrials.gov/ct2/results?term=exosomes. (accessed March 30, 2020).

[B52] Colson Y. L. Grinstaff M. W. (2012). Biologically responsive polymeric nanoparticles for drug delivery. *Adv. Mater.* 24 3878–3886. 10.1002/adma.201200420 22988558

[B53] Croissant J. G. Fatieiev Y. Almalik A. Khashab N. M. (2018). Mesoporous silica and organosilica nanoparticles: physical chemistry, biosafety, delivery strategies, and biomedical applications. *Adv. Healthc Mater* 7:831. 10.1002/adhm.201700831 29193848

[B54] Crucho C. I. C. Barros M. T. (2017). Polymeric nanoparticles: a study on the preparation variables and characterization methods. *Mater Sci. Eng. C Mater Biol. Appl.* 80 771–784. 10.1016/j.msec.2017.06.004 28866227

[B55] Dadfar S. M. Camozzi D. Darguzyte M. Roemhild K. Varvara P. Metselaar J. (2020). Size-isolation of superparamagnetic iron oxide nanoparticles improves MRI, MPI and hyperthermia performance. *J. Nanobiotechnol.* 18:22. 10.1186/s12951-020-0580-1 31992302 PMC6986086

[B56] Darbey A. Smith L. B. (2018). Deliverable transgenics & gene therapy possibilities for the testes. *Mol. Cell Endocrinol.* 468 81–94. 10.1016/j.mce.2017.11.023 29191697

[B57] Das C. K. Jena B. C. Banerjee I. Das S. Parekh A. Bhutia S. K. (2019). Exosome as a novel shuttle for delivery of therapeutics across biological barriers. *Mol. Pharm.* 16 24–40. 10.1021/acs.molpharmaceut.8b00901 30513203

[B58] Deng Z. Wang N. Ai F. Wang Z. Zhu G. (2020). Nanomaterial-mediated platinum drug-based combinatorial cancer therapy. *View* 2:30. 10.1002/viw.20200030

[B59] Devine P. J. Perreault S. D. Luderer U. (2012). Roles of reactive oxygen species and antioxidants in ovarian toxicity. *Biol. Reprod.* 86:27. 10.1095/biolreprod.111.095224 22034525 PMC3290661

[B60] Du D. Fu H. J. Ren W. W. Li X. L. Guo L. H. (2020). PSA targeted dual-modality manganese oxide-mesoporous silica nanoparticles for prostate cancer imaging. *Biomed. Pharmacother.* 121:109614. 10.1016/j.biopha.2019.109614 31731188

[B61] Durfey C. L. Swistek S. E. Liao S. F. Crenshaw M. A. Clemente H. J. Thirumalai R. (2019). Nanotechnology-based approach for safer enrichment of semen with best spermatozoa. *J. Anim. Sci. Biotechnol.* 10:14. 10.1186/s40104-018-0307-4 30774950 PMC6368687

[B62] Ebrahimi N. Bi C. Cappelleri D. J. Ciuti G. Conn A. T. Faivre D. (2020). Magnetic actuation methods in bio/soft robotics. *Adv. Funct. Mater.* 31:2005137. 10.1002/adfm.202005137

[B63] Ehlerding E. B. Chen F. Cai W. (2016). Biodegradable and renal clearable inorganic nanoparticles. *Adv. Sci.* 3:223. 10.1002/advs.201500223 27429897 PMC4944857

[B64] El-Sayed A. Kamel M. (2020). Advanced applications of nanotechnology in veterinary medicine. *Environ. Sci. Pollut. Res. Int.* 27 19073–19086. 10.1007/s11356-018-3913-y 30547342

[B65] El-Shabasy R. M. Farouk Elsadek M. Mohamed Ahmed B. Fawzy Farahat M. Mosleh K. N. Taher M. M. (2021). Recent developments in carbon quantum dots: properties, fabrication techniques, and bio-applications. *Processes* 9:388. 10.3390/pr9020388

[B66] Ema M. Okuda H. Gamo M. Honda K. (2017). A review of reproductive and developmental toxicity of silver nanoparticles in laboratory animals. *Reprod Toxicol.* 67 149–164. 10.1016/j.reprotox.2017.01.005 28088501

[B67] Evans T. J. Ganjam V. K. (2011). “Reproductive anatomy and physiology,” in *Reproductive and Developmental Toxicology*, ed. Gupta R. C. 7–32.

[B68] Falchi L. Khalil W. A. Hassan M. Marei W. F. A. (2018). Perspectives of nanotechnology in male fertility and sperm function. *Int. J. Vet. Sci. Med.* 6 265–269. 10.1016/j.ijvsm.2018.09.001 30564607 PMC6286411

[B69] Fan L. Chen J. Zhang X. Liu Y. Xu C. (2014). Follicle-stimulating hormone polypeptide modified nanoparticle drug delivery system in the treatment of lymphatic metastasis during ovarian carcinoma therapy. *Gynecol. Oncol.* 135 125–132. 10.1016/j.ygyno.2014.06.030 25003656

[B70] Fang Y. Lin S. Yang F. Situ J. Lin S. Luo Y. (2020). Aptamer-conjugated multifunctional polymeric nanoparticles as cancer-targeted, MRI-ultrasensitive drug delivery systems for treatment of castration-resistant prostate cancer. *Biomed. Res. Int.* 2020:9186583. 10.1155/2020/9186583 32420382 PMC7201588

[B71] Farini V. L. Camano C. V. Ybarra G. Viale D. L. Vichera G. Yakisich J. S. (2016). Improvement of bovine semen quality by removal of membrane-damaged sperm cells with DNA aptamers and magnetic nanoparticles. *J. Biotechnol.* 229 33–41. 10.1016/j.jbiotec.2016.05.008 27164256

[B72] Fathi M. Barar J. Erfan-Niya H. Omidi Y. (2020). Methotrexate-conjugated chitosan-grafted pH- and thermo-responsive magnetic nanoparticles for targeted therapy of ovarian cancer. *Int. J. Biol. Macromol.* 154 1175–1184. 10.1016/j.ijbiomac.2019.10.272 31730949

[B73] Feugang J. M. Liao S. F. Crenshaw M. A. Clemente H. Willard S. T. Ryan P. L. (2015a). Lectin-functionalized magnetic iron oxide nanoparticles for reproductive improvement. *J. Fertil. Vit. IVF-Worldwide Reprodu. Med. Genet. Stem Cell* 3:145. 10.4172/2375-4508.1000145

[B74] Feugang J. M. Youngblood R. C. Greene J. M. Fahad A. S. Monroe W. A. Willard S. T. (2012). Application of quantum dot nanoparticles for potential non-invasive bio-imaging of mammalian spermatazoa. *J. Nanobiotechnol.* 10:45. 10.1186/1477-3155-10-45 23241497 PMC3553073

[B75] Feugang J. M. Youngblood R. C. Greene J. M. Willard S. T. Ryan P. L. (2015b). Self-illuminating quantum dots for non-invasive bioluminescence imaging of mammalian gametes. *J. Nanobiotechnol.* 13:38. 10.1186/s12951-015-0097-1 26040273 PMC4455054

[B76] Feugang J. M. Rhoads C. E. Mustapha P. A. Tardif S. Parrish J. J. Willard S. T. (2019). Treatment of boar sperm with nanoparticles for improved fertility. *Theriogenology* 137 75–81. 10.1016/j.theriogenology.2019.05.040 31204016

[B77] Ghoreifi A. Djaladat H. (2019). Management of primary testicular tumor. *Urol. Clin. North Am.* 46 333–339. 10.1016/j.ucl.2019.04.006 31277728

[B78] Gilligan K. E. Dwyer R. M. (2017). Engineering exosomes for cancer therapy. *Int. J. Mol. Sci.* 18:1122. 10.3390/ijms18061122 28538671 PMC5485946

[B79] Goncalves D. R. Leroy J. Van Hees S. Xhonneux I. Bols P. E. J. Kiekens F. (2020). Cellular uptake of polymeric nanoparticles by bovine cumulus-oocyte complexes and their effect on in vitro developmental competence. *Eur. J. Pharm. Biopharm.* 158 143–155. 10.1016/j.ejpb.2020.11.011 33248266

[B80] Gregoriadis G. Ryman B. E. (1971). Liposomes as carriers of enzymes or drugs: a new approach to the treatment of storage diseases. *Biochem. J.* 124:58. 10.1042/bj1240058p PMC1177319 5130994

[B81] Habas K. Demir E. Guo C. Brinkworth M. H. Anderson D. (2021). Toxicity mechanisms of nanoparticles in the male reproductive system. *Drug Metab. Rev.* 1–14. 10.1080/03602532.2021.1917597 33989097

[B82] Halliwell B. (1991). Reactive oxygen species in living systems: source, biochemistry, and role in human disease. *Am. J. Med.* 91, 14S–22S. 10.1016/0002-9343(91)90279-7 1928205

[B83] Hanoux V. Pairault C. Bakalska M. Habert R. Livera G. (2007). Caspase-2 involvement during ionizing radiation-induced oocyte death in the mouse ovary. *Cell Death Differ.* 14, 671–681. 10.1038/sj.cdd.4402052 17082817

[B84] Hermo L. Pelletier R. M. Cyr D. G. Smith C. E. (2010). Surfing the wave, cycle, life history, and genes/proteins expressed by testicular germ cells. part 1: background to *Spermatogenesis*,spermatogonia, and spermatocytes. *Microsc. Res. Tech.* 73 241–278. 19941293 10.1002/jemt.20783

[B85] Hong S. S. Zhang M. X. Zhang M. Yu Y. Chen J. Zhang X. Y. (2018). Follicle-stimulating hormone peptide-conjugated nanoparticles for targeted shRNA delivery lead to effective gro-alpha silencing and antitumor activity against ovarian cancer. *Drug Deliv.* 25 576–584. 10.1080/10717544.2018.1440667 29461120 PMC6058603

[B86] Hoog J. L. Lotvall J. (2015). Diversity of extracellular vesicles in human ejaculates revealed by cryo-electron microscopy. *J. Extr. Vesicles* 4:28680. 10.3402/jev.v4.28680 26563734 PMC4643196

[B87] Hoyer P. B. Devine P. J. Hu X. Thompson K. E. Sipes G. (2001). Ovarian toxicity of 4-vinylcyclohexene diepoxide: a mechanistic model. *Toxicol. Pathol.* 29, 91–99. 11215690 10.1080/019262301301418892

[B88] Hu C. M. Zhang L. Aryal S. Cheung C. Fang R. H. Zhang L. (2011). Erythrocyte membrane-camouflaged polymeric nanoparticles as a biomimetic delivery platform. *Proc. Natl. Acad. Sci. U.S.A.* 108 10980–10985. 10.1073/pnas.1106634108 21690347 PMC3131364

[B89] Huang D.-M. Hung Y. Ko B.-S. Hsu S.-C. Chen W.-H. Chien C.-L. (2005). Highly efficient cellular labeling of mesoporous nanoparticles in human mesenchymal stem cells: implication for stem cell tracking. *FASEB* 19 2014–2016. 10.1096/fj.05-4288fje 16230334

[B90] Imran M. Saleem S. Chaudhuri A. Ali J. Baboota S. (2020). Docetaxel: an update on its molecular mechanisms, therapeutic trajectory and nanotechnology in the treatment of breast, lung and prostate cancer. *J. Drug Deliv. Sci. Technol.* 60:101959. 10.1016/j.jddst.2020.101959

[B91] Inhorn M. C. Patrizio P. (2015). Infertility around the globe: new thinking on gender, reproductive technologies and global movements in the 21st century. *Hum. Reprod Update* 21 411–426. 10.1093/humupd/dmv016 25801630

[B92] Iravani S. Varma R. S. (2020). Green synthesis, biomedical and biotechnological applications of carbon and graphene quantum dots. A review. *Environ. Chem. Lett.* 18, 703–727. 10.1007/s10311-020-00984-0 32206050 PMC7088420

[B93] Jafari S. Derakhshankhah H. Alaei L. Fattahi A. Varnamkhasti B. S. Saboury A. A. (2019). Mesoporous silica nanoparticles for therapeutic/diagnostic applications. *Biomed. Pharmacother.* 109 1100–1111. 10.1016/j.biopha.2018.10.167 30551360

[B94] Jamaludin N. A. Thurston L. M. Witek K. J. Meikle A. Basatvat S. Elliott S. (2019). Efficient isolation, biophysical characterisation and molecular composition of extracellular vesicles secreted by primary and immortalised cells of reproductive origin. *Theriogenology* 135 121–137. 10.1016/j.theriogenology.2019.06.002 31207473

[B95] Kaur G. Mital P. Dufour J. M. (2013). Testisimmune privilegeassumptions versus facts. *Animal Reprodu.* 10 3–15. PMC4192663 25309630

[B96] Khalil I. S. M. Magdanz V. Simmchen J. Klingner A. Misra S. (2020). Resemblance between motile and magnetically actuated sperm cells. *Appl. Phys. Lett.* 116:063702. 10.1063/1.5142470

[B97] Kharazi U. Badalzadeh R. (2020). A review on the stem cell therapy and an introduction to exosomes as a new tool in reproductive medicine. *Reprod Biol.* 20 447–459. 10.1016/j.repbio.2020.07.002 32900639

[B98] Kim O. Y. Lee J. Gho Y. S. (2017). Extracellular vesicle mimetics: novel alternatives to extracellular vesicle-based theranostics, drug delivery, and vaccines. *Semin. Cell Dev. Biol.* 67 74–82. 10.1016/j.semcdb.2016.12.001 27916566

[B99] Kim W. J. Kim B. S. Kim H. J. Cho Y. D. Shin H. L. Yoon H. I. (2020). Intratesticular peptidyl prolyl isomerase 1 protein delivery using cationic lipid-coated fibroin nanoparticle complexes rescues male infertility in mice. *ACS Nano.* 14 13217–13231. 10.1021/acsnano.0c04936 32969647

[B100] Koo O. M. Rubinstein I. Onyuksel H. (2005). Role of nanotechnology in targeted drug delivery and imaging: a concise review. *Nanomedicine* 1 193–212. 10.1016/j.nano.2005.06.004 17292079

[B101] Kumari A. Yadav S. K. Yadav S. C. (2010). Biodegradable polymeric nanoparticles based drug delivery systems. *Colloids Surf B Biointerfaces* 75 1–18. 10.1016/j.colsurfb.2009.09.001 19782542

[B102] Lakkireddy H. R. Bazile D. (2016). Building the design, translation and development principles of polymeric nanomedicines using the case of clinically advanced poly(lactide(glycolide))-poly(ethylene glycol) nanotechnology as a model: an industrial viewpoint. *Adv. Drug Deliv. Rev.* 107 289–332. 10.1016/j.addr.2016.08.012 27593265

[B103] Le Joncour V. Laakkonen P. (2018). Seek & destroy, use of targeting peptides for cancer detection and drug delivery. *Bioorg Med. Chem.* 26 2797–2806. 10.1016/j.bmc.2017.08.052 28893601

[B104] Li K. Zhan W. Chen Y. Jha R. K. Chen X. (2019). Docetaxel and doxorubicin codelivery by nanocarriers for synergistic treatment of prostate cancer. *Front. Pharmacol.* 10:1436. 10.3389/fphar.2019.01436 31920642 PMC6930690

[B105] Li W. Fu J. Zhang S. Zhao J. Xie N. Cai G. (2015). The proteasome inhibitor bortezomib induces testicular toxicity by upregulation of oxidative stress, AMP-activated protein kinase (AMPK) activation and deregulation of germ cell development in adult murine testis. *Toxicol. Appl. Pharmacol.* 285, 98–109. 10.1016/j.taap.2015.04.001 25886977

[B106] Li Y. Gao Y. Zhang X. Guo H. Gao H. (2020). Nanoparticles in precision medicine for ovarian cancer: from chemotherapy to immunotherapy. *Int. J. Pharm.* 591:119986. 10.1016/j.ijpharm.2020.119986 33069895

[B107] Liao C. Li Y. Tjong S. C. (2019). Bactericidal and cytotoxic properties of silver nanoparticles. *Int. J. Mol. Sci.* 20:449. 10.3390/ijms20020449 30669621 PMC6359645

[B108] Lian H. Wu J. Hu Y. Guo H. (2017). Self-assembled albumin nanoparticles for combination therapy in prostate cancer. *Int. J. Nanomed.* 12 7777–7787. 10.2147/IJN.S144634 29123392 PMC5661507

[B109] Lin Z. Monteiro-Riviere N. A. Riviere J. E. (2015). Pharmacokinetics of metallic nanoparticles. *Wiley Interdiscip Rev. Nano. Nanobiotechnol.* 7 189–217. 10.1002/wnan.1304 25316649

[B110] Liu C. Su C. (2019). Design strategies and application progress of therapeutic exosomes. *Theranostics* 9 1015–1028. 10.7150/thno.30853 30867813 PMC6401399

[B111] Liu D. Bimbo L. M. Makila E. Villanova F. Kaasalainen M. Herranz-Blanco B. (2013). Co-delivery of a hydrophobic small molecule and a hydrophilic peptide by porous silicon nanoparticles. *J. Control Release* 170 268–278. 10.1016/j.jconrel.2013.05.036 23756152

[B112] Liu J. Liu T. Pan J. Liu S. Lu G. Q. M. (2018). Advances in Multicompartment mesoporous silica micro/nanoparticles for theranostic applications. *Annu. Rev. Chem. Biomol. Eng.* 9 389–411. 10.1146/annurev-chembioeng-060817-084225 29618224

[B113] Liu N. Ji J. Qiu H. Shao Z. Wen X. Chen A. (2020). Improving radio-chemotherapy efficacy of prostate cancer by co-deliverying docetaxel and dbait with biodegradable nanoparticles. *Artif Cells Nano. Biotechnol.* 48 305–314. 10.1080/21691401.2019.1703726 31858836

[B114] Liu N. Tang M. (2020). Toxicity of different types of quantum dots to mammalian cells *in vitro*: an update review. *J. Hazard. Mater.* 399:122606. 10.1016/j.jhazmat.2020.122606 32516645

[B115] Longcore T. Rich C. Sullivan L. M. (2009). Critical assessment of claims regarding management of feral cats by trap-neuter-return. *Conserv Biol.* 23 887–894. 10.1111/j.1523-1739.2009.01174.x 19245489

[B116] Lorenc T. Klimczyk K. Michalczewska I. Slomka M. Kubiak-Tomaszewska G. Olejarz W. (2020). Exosomes in prostate cancer diagnosis, prognosis and therapy. *Int. J. Mol. Sci.* 21:118. 10.3390/ijms21062118 32204455 PMC7139716

[B117] Lu M. Huang Y. (2020). Bioinspired exosome-like therapeutics and delivery nanoplatforms. *Biomaterials* 242:119925. 10.1016/j.biomaterials.2020.119925 32151860

[B118] Magdanz V. Khalil I. S. M. Simmchen J. Furtado M. H. Mohanty S. Gebauer J. (2020). IRONsperm: sperm-templated soft robotic micrrobots. *Sci. Adv.* 6:eaba5855. 10.1126/sciadv.aba5855 32923590 PMC7450605

[B119] Magdanz V. Vivaldi J. Mohanty S. Klingner A. Vendittelli M. Simmchen J. (2021). Impact of segmented magnetization on the flagellar propulsion of sperm-templated microrobots. *Adv. Sci.* 8:2004037. 10.1002/advs.202004037 33898186 PMC8061355

[B120] Manzano M. Vallet-Regí M. (2019). Mesoporous silica nanoparticles for drug delivery. *Adv. Funct. Mater.* 30:1902634. 10.1002/adfm.201902634

[B121] Marzano G. Chiriaco M. S. Primiceri E. Dell’Aquila M. E. Ramalho-Santos J. Zara V. (2020). Sperm selection in assisted reproduction: a review of established methods and cutting-edge possibilities. *Biotechnol. Adv.* 40:107498. 10.1016/j.biotechadv.2019.107498 31836499

[B122] Masood F. (2016). Polymeric nanoparticles for targeted drug delivery system for cancer therapy. *Mater Sci. Eng. C Mater Biol. Appl.* 60 569–578. 10.1016/j.msec.2015.11.067 26706565

[B123] Meng H. Leong W. Leong K. W. Chen C. Zhao Y. (2018). Walking the line: the fate of nanomaterials at biological barriers. *Biomaterials* 174 41–53. 10.1016/j.biomaterials.2018.04.056 29778981 PMC5984195

[B124] Mitchell M. J. Billingsley M. M. Haley R. M. Wechsler M. E. Peppas N. A. Langer R. (2021). Engineering precision nanoparticles for drug delivery. *Nat. Rev. Drug Discov.* 20, 101–124. 10.1038/s41573-020-0090-8 33277608 PMC7717100

[B125] Mohajeri M. Bianconi V. Avila-Rodriguez M. F. Barreto G. E. Jamialahmadi T. Pirro M. (2020). Curcumin: a phytochemical modulator of estrogens and androgens in tumors of the reproductive system. *Pharmacol. Res.* 156:104765. 10.1016/j.phrs.2020.104765 32217147

[B126] Mohanta D. Patnaik S. Sood S. Das N. (2019). Carbon nanotubes: evaluation of toxicity at biointerfaces. *J. Pharm. Anal.* 9, 293–300. 10.1016/j.jpha.2019.04.003 31929938 PMC6951486

[B127] Morishita Y. Yoshioka Y. Satoh H. Nojiri N. Nagano K. Abe Y. (2012). Distribution and histologic effects of intravenously administered amorphous nanosilica particles in the testes of mice. *Biochem. Biophys. Res. Commun.* 420 297–301. 10.1016/j.bbrc.2012.02.153 22417826

[B128] Mruk D. D. Wong C. H. Silvestrini B. Cheng C. Y. (2006). A male contraceptive targeting germ cell adhesion. *Nat. Med.* 12 1323–1328. 10.1038/nm1420 17072312

[B129] Nguyen K. C. Zhang Y. Todd J. Kittle K. Lalande M. Smith S. (2020). Hepatotoxicity of cadmium telluride quantum dots induced by mitochondrial dysfunction. *Chem. Res. Toxicol.* 33, 2286–2297. 10.1021/acs.chemrestox.9b00526 32844644

[B130] Niederberger C. Pellicer A. Cohen J. Gardner D. K. Palermo G. D. O’Neill C. L. (2018). Forty years of IVF. *Fertil Steril* 18:e185. 10.1016/j.fertnstert.2018.06.005 30053940

[B131] Niu W. Wang J. Wang Q. Shen J. (2020). Celastrol loaded nanoparticles with ROS-response and ROS-inducer for the treatment of ovarian cancer. *Front. Chem.* 8:574614. 10.3389/fchem.2020.574614 33195064 PMC7662441

[B132] Noorani L. Stenzel M. Liang R. Pourgholami M. H. Morris D. L. (2015). Albumin nanoparticles increase the anticancer efficacy of albendazole in ovarian cancer xenograft model. *J. Nanobiotechnol.* 13:25. 10.1186/s12951-015-0082-8 25890381 PMC4409778

[B133] O’Flaherty C. Matsushita-Fournier D. (2017). Reactive oxygen species and protein modifications in spermatozoa. *Biol. Reprod.* 97, 577–585. 10.1093/biolre/iox104 29025014

[B134] Oltra N. S. Nair P. Discher D. E. (2014). From stealthy polymersomes and filomicelles to “self” Peptide-nanoparticles for cancer therapy. *Annu. Rev. Chem. Biomol. Eng.* 5 281–299. 10.1146/annurev-chembioeng-060713-040447 24910917 PMC4387849

[B135] O’Neil E. V. Burns G. W. Spencer T. E. (2020). Extracellular vesicles: Novel regulators of conceptus-uterine interactions? *Theriogenology* 150 106–112. 10.1016/j.theriogenology.2020.01.083 32164992 PMC8559595

[B136] Pacheco A. Blanco A. Bronet F. Cruz M. Garcia-Fernandez J. Garcia-Velasco J. A. (2020). Magnetic-activated cell sorting (MACS): a useful sperm-selection technique in cases of high levels of sperm DNA fragmentation. *J. Clin. Med.* 9:976. 10.3390/jcm9123976 33302575 PMC7763893

[B137] Pagseesing S. Yostawonkul J. Surassmo S. Boonrungsiman S. Namdee K. Khongkow M. (2018). Formulation, physical, in vitro and ex vivo evaluation of nanomedicine-based chemosterilant for non-surgical castration of male animals. *Theriogenology* 108 167–175. 10.1016/j.theriogenology.2017.12.014 29223654

[B138] Paktinat S. Hashemi S. M. Ghaffari Novin M. Mohammadi-Yeganeh S. Salehpour S. Karamian A. (2019). Seminal exosomes induce interleukin-6 and interleukin-8 secretion by human endometrial stromal cells. *Eur. J. Obstet. Gynecol. Reprod Biol.* 235 71–76. 10.1016/j.ejogrb.2019.02.010 30807994

[B139] Pecot C. V. Calin G. A. Coleman R. L. Lopez-Berestein G. Sood A. K. (2011). RNA interference in the clinic: challenges and future directions. *Nat. Rev. Cancer* 11 59–67. 10.1038/nrc2966 21160526 PMC3199132

[B140] Peer D. Karp J. M. Hong S. Farokhzad O. C. Margalit R. Langer R. (2007). Nanocarriers as an emerging platform for cancer therapy. *Nat. Nanotechnol.* 2 751–760. 18654426 10.1038/nnano.2007.387

[B141] Pietroiusti A. Campagnolo L. Fadeel B. (2013). Interactions of engineered nanoparticles with organs protected by internal biological barriers. *Small* 9 1557–1572. 10.1002/smll.201201463 23097249

[B142] Poniatowska G. Michalski W. Kucharz J. Jonska-Gmyrek J. Wieszczy P. Nietupski K. (2020). What is the damage? Testicular germ cell tumour survivors deficient in testosterone at risk of metabolic syndrome and a need for medical intervention. *Med. Oncol.* 37:82. 10.1007/s12032-020-01407-4 32767179

[B143] Prajapati S. K. Malaiya A. Kesharwani P. Soni D. Jain A. (2020). Biomedical applications and toxicities of carbon nanotubes. *Drug Chem. Toxicol.* 1–16. 10.1080/01480545.2019.1709492 31908176

[B144] Pullan J. E. Confeld M. I. Osborn J. K. Kim J. Sarkar K. Mallik S. (2019). Exosomes as drug carriers for cancer therapy. *Mol. Pharm.* 16 1789–1798. 10.1021/acs.molpharmaceut.9b00104 30951627

[B145] Punab M. Poolamets O. Paju P. Vihljajev V. Pomm K. Ladva R. (2017). Causes of male infertility: a 9-year prospective monocentre study on 1737 patients with reduced total sperm counts. *Hum. Reprod* 32 18–31. 10.1093/humrep/dew284 27864361 PMC5165077

[B146] Raimondo S. Gentile T. Gentile M. Donnarumma F. Esposito G. Morelli A. (2020). Comparing different sperm separation techniques for ART, through quantitative evaluation of p53 protein. *J. Hum. Reprod. Sci.* 13, 117–124. 10.4103/jhrs.JHRS_117_19 32792760 PMC7394090

[B147] Ramot Y. Haim-Zada M. Domb A. J. Nyska A. (2016). Biocompatibility and safety of PLA and its copolymers. *Adv. Drug Deliv. Rev.* 107 153–162. 10.1016/j.addr.2016.03.012 27058154

[B148] Raoofi A. Delbari A. Mahdian D. Mojadadi M. S. Akhlaghi M. Dadashizadeh G. (2020). Effects of curcumin nanoparticle on the histological changes and apoptotic factors expression in testis tissue after methylphenidate administration in rats. *Acta Histochem* 123:151656. 10.1016/j.acthis.2020.151656 33249311

[B149] Rateb S. A. (2020). Purification of cryopreserved camel spermatozoa following protease-based semen liquefaction by lectin-functionalized DNA-defrag magnetic nanoparticles. *Reprod Domest Anim.* 56 183–192. 10.1111/rda.13863 33170990

[B150] Rebourcet D. O’Shaughnessy P. J. Monteiro A. Milne L. Cruickshanks L. Jeffrey N. (2014). Sertoli cells maintain leydig cell number and peritubular myoid cell activity in the adult mouse testis. *PLoS One* 9:e105687. 10.1371/journal.pone.0105687 25144714 PMC4140823

[B151] Rodriguez P. L. Harada T. Christian D. A. Pantano D. A. Tsai R. K. Discher D. E. (2013). Minimal “Self” peptides that inhibit phagocytic clearance and enhance delivery of nanoparticles. *Science* 339 971–975. 10.1126/science.1229568 23430657 PMC3966479

[B152] Rodriguez-Caro H. Dragovic R. Shen M. Dombi E. Mounce G. Field K. (2019). In vitro decidualisation of human endometrial stromal cells is enhanced by seminal fluid extracellular vesicles. *J. Extracell Vesicles* 8:1565262. 10.1080/20013078.2019.1565262 30728921 PMC6352950

[B153] Ruoslahti E. (2012). Peptides as targeting elements and tissue penetration devices for nanoparticles. *Adv. Mater* 24 3747–3756. 10.1002/adma.201200454 22550056 PMC3947925

[B154] Saadeldin I. M. Khalil W. A. Alharbi M. G. Lee S. H. (2020). The current trends in using nanoparticles, liposomes, and exosomes for semen cryopreservation. *Animals* 10:281. 10.3390/ani10122281 33287256 PMC7761754

[B155] Sabio R. M. Meneguin A. B. Ribeiro T. C. Silva R. R. Chorilli M. (2019). New insights towards mesoporous silica nanoparticles as a technological platform for chemotherapeutic drugs delivery. *Int. J. Pharm* 564 379–409. 10.1016/j.ijpharm.2019.04.067 31028801

[B156] Sanchez-Martin P. Dorado-Silva M. Sanchez-Martin F. Gonzalez Martinez M. Johnston S. D. Gosalvez J. (2017). Magnetic cell sorting of semen containing spermatozoa with high DNA fragmentation in ICSI cycles decreases miscarriage rate. *Reprod. Biomed. Online* 34, 506–512. 10.1016/j.rbmo.2017.01.015 28283446

[B157] Sanocka D. Kurpisz M. (2004). Reactive oxygen species and sperm cells. *Reprod. Biol. Endocrinol.* 2:12. 10.1186/1477-7827-2-12 15038829 PMC400757

[B158] Saradha B. Mathur P. P. (2006). Effect of environmental contaminants on male reproduction. *Environ. Toxicol. Pharmacol.* 21, 34–41. 10.1016/j.etap.2005.06.004 21783636

[B159] Sasidharan A. Monteiro-Riviere N. A. (2015). Biomedical applications of gold nanomaterials: opportunities and challenges. *Wiley Interdiscip. Rev. Nanomed. Nanobiotechnol.* 7, 779–796. 10.1002/wnan.1341 25808787

[B160] Sebesta E. M. Anderson C. B. (2017). The surgical management of prostate cancer. *Semin Oncol.* 44 347–357. 10.1053/j.seminoncol.2018.01.003 29580436

[B161] Sercombe L. Veerati T. Moheimani F. Wu S. Y. Sood A. K. Hua S. (2015). Advances and challenges of liposome assisted drug delivery. *Front. Pharmacol.* 6:286. 10.3389/fphar.2015.00286 26648870 PMC4664963

[B162] Shabani R. Ashjari M. Ashtari K. Izadyar F. Behnam B. Khoei S. (2018). Elimination of mouse tumor cells from neonate spermatogonial cells utilizing cisplatin-entrapped folic acid-conjugated poly(lactic-co-glycolic acid) nanoparticles in vitro. *Int. J. Nanomed.* 13 2943–2954. 10.2147/IJN.S155052 29849458 PMC5965374

[B163] Shandilya R. Pathak N. Lohiya N. K. Sharma R. S. Mishra P. K. (2020). Nanotechnology in reproductive medicine: opportunities for clinical translation. *Clin. Exp. Reprod Med.* 47 245–262. 10.5653/cerm.2020.03650 33227186 PMC7711096

[B164] Sharpe R. M. Maddocks S. Kerr J. B. (1990). Cell-cell interactions in the control of spermatogenesis as studied using leydig cell destruction and testosterone repacement. *Am. J. Anat.* 188 3–20. 2161173 10.1002/aja.1001880103

[B165] Shimizu A. Kawashima S. (1989). Kinetic study of internalization and degradation of 131I-labeled follicle-stimulating hormone in mouse sertoli cells and its relevance to other systems. *J. Biol. Chem.* 264 13632–13638. 2503503

[B166] Shimizu A. Tsutsui K. Kawashima S. (1987). Autoradiographic study of binding and internalization of follicle-stimulating hormone in the mouse testis minces in vitro. *Endocrinol. Japan* 34 431–442. 3115766 10.1507/endocrj1954.34.431

[B167] Skotland T. Sandvig K. Llorente A. (2017). Lipids in exosomes: current knowledge and the way forward. *Prog. Lipid Res.* 66 30–41. 10.1016/j.plipres.2017.03.001 28342835

[B168] Song Y. Y. Yuan Y. Shi X. Che Y. Y. (2020). Improved drug delivery and anti-tumor efficacy of combinatorial liposomal formulation of genistein and plumbagin by targeting Glut1 and Akt3 proteins in mice bearing prostate tumor. *Colloids Surf B Biointerfaces* 190:110966. 10.1016/j.colsurfb.2020.110966 32199263

[B169] Stanton P. G. (2016). Regulation of the blood-testis barrier. *Semin Cell Dev. Biol.* 59 166–173. 10.1016/j.semcdb.2016.06.018 27353840

[B170] Stueber D. D. Villanova J. Aponte I. Xiao Z. Colvin V. L. (2021). Magnetic nanoparticles in biology and medicine: past, present, and future trends. *Pharmaceutics* 13:943. 10.3390/pharmaceutics13070943 34202604 PMC8309177

[B171] Stylianopoulos T. Jain R. K. (2015). Design considerations for nanotherapeutics in oncology. *Nanomedicine* 11 1893–1907. 10.1016/j.nano.2015.07.015 26282377 PMC4628869

[B172] Sullivan R. (2015). Epididymosomes: a heterogeneous population of microvesicles with multiple functions in sperm maturation and storage. *Asian J. Androl.* 17 726–729. 10.4103/1008-682X.155255 26112475 PMC4577580

[B173] Sullivan R. Frenette G. Girouard J. (2007). Epididymosomes are involved in the acquisition of new sperm proteins during epididymal transit. *Asian J. Androl.* 9 483–491. 10.1111/j.1745-7262.2007.00281.x 17589785

[B174] Tesfaye D. Hailay T. Salilew-Wondim D. Hoelker M. Bitseha S. Gebremedhn S. (2020). Extracellular vesicle mediated molecular signaling in ovarian follicle: implication for oocyte developmental competence. *Theriogenology* 150 70–74. 10.1016/j.theriogenology.2020.01.075 32088041

[B175] Tompkins A. J. Chatterjee D. Maddox M. Wang J. Arciero E. Camussi G. (2015). The emergence of extracellular vesicles in urology: fertility, cancer, biomarkers and targeted pharmacotherapy. *J. Extracell Vesicles* 4:23815. 10.3402/jev.v4.23815 26134460 PMC4488336

[B176] Tran P. H. L. Xiang D. Tran T. T. D. Yin W. Zhang Y. Kong L. (2019). Exosomes and nanoengineering: a match made for precision therapeutics. *Adv. Mater* 32:e1904040. 10.1002/adma.201904040 31531916

[B177] Trigg N. A. Eamens A. L. Nixon B. (2019). The contribution of epididymosomes to the sperm small RNA profile. *Reproduction* 157 R209–R223. 10.1530/REP-18-0480 30780129

[B178] Uzun B. Atli O. Perk B. O. Burukoglu D. Ilgin S. (2015). Evaluation of the reproductive toxicity of naproxen sodium and meloxicam in male rats. *Hum. Exp. Toxicol.* 34, 415–429. 10.1177/0960327114542886 25034942

[B179] van Niel G. D’Angelo G. Raposo G. (2018). Shedding light on the cell biology of extracellular vesicles. *Nat. Rev. Mol. Cell. Biol.* 19 213–228. 10.1038/nrm.2017.125 29339798

[B180] Vasquez E. S. Feugang J. M. Willard S. T. Ryan P. L. Walters K. B. (2016). Bioluminescent magnetic nanoparticles as potential imaging agents for mammalian spermatozoa. *J. Nanobiotechnol.* 14:20. 10.1186/s12951-016-0168-y 26984640 PMC4794913

[B181] Vassal M. Rebelo S. Pereira M. L. (2021). Metal oxide nanoparticles: evidence of adverse effects on the male reproductive system. *Int. J. Mol. Sci.* 22:8061. 10.3390/ijms22158061 34360825 PMC8348343

[B182] Vermeulen M. Poels J. de Michele F. des Rieux A. Wyns C. (2017). Restoring fertility with cryopreserved prepubertal testicular tissue: perspectives with hydrogel encapsulation, nanotechnology, and bioengineered scaffolds. *Ann. Biomed. Eng.* 45 1770–1781. 10.1007/s10439-017-1789-5 28070774

[B183] Von Hoff D. D. Mita M. M. Ramanathan R. K. Weiss G. J. Mita A. C. LoRusso P. M. (2016). Phase I study of PSMA-targeted docetaxel-containing nanoparticle BIND-014 in patients with advanced solid tumors. *Clin. Cancer Res.* 22 3157–3163. 10.1158/1078-0432.CCR-15-2548 26847057

[B184] Walcher L. Kistenmacher A. K. Suo H. Kitte R. Dluczek S. Strauss A. (2020). Cancer stem cells-origins and biomarkers: perspectives for targeted personalized therapies. *Front. Immunol.* 11:1280. 10.3389/fimmu.2020.01280 32849491 PMC7426526

[B185] Wan Z. Xie F. Wang L. Zhang G. Zhang H. (2020). Preparation and evaluation of cabazitaxel-loaded bovine serum albumin nanoparticles for prostate cancer. *Int. J. Nanomed.* 15 5333–5344. 10.2147/IJN.S258856 32801692 PMC7402868

[B186] Wang W. Craig Z. R. Basavarajappa M. S. Gupta R. K. Flaws J. A. (2012). Di (2-ethylhexyl) phthalate inhibits growth of mouse ovarian antral follicles through an oxidative stress pathway. *Toxicol. Appl. Pharmacol.* 258, 288–295. 10.1016/j.taap.2011.11.008 22155089 PMC3259146

[B187] Wolfram J. Zhu M. Yang Y. Shen J. Gentile E. Paolino D. (2015). Safety of nanoparticles in medicine. *Curr. Drug Targets* 16 1671–1681. 26601723 10.2174/1389450115666140804124808 PMC4964712

[B188] Woodle M. C. Lasic D. D. (1992). Sterically stabilised liposomes. *Biochim. et Biophys. Acta* 1113 171–199. 10.1016/0304-4157(92)90038-c 1510996

[B189] Wu C. H. Liu I. J. Lu R. M. Wu H. C. (2016). Advancement and applications of peptide phage display technology in biomedical science. *J. Biomed. Sci.* 23:8. 10.1186/s12929-016-0223-x 26786672 PMC4717660

[B190] Xie P. Yang S. T. He T. Yang S. Tang X. H. (2017). Bioaccumulation and toxicity of carbon nanoparticles suspension injection in intravenously exposed mice. *Int. J. Mol. Sci.* 18:2562. 10.3390/ijms18122562 29186019 PMC5751165

[B191] Yostawonkul J. Surassmo S. Namdee K. Khongkow M. Boonthum C. Pagseesing S. (2017). Nanocarrier-mediated delivery of alpha-mangostin for non-surgical castration of male animals. *Sci. Rep.* 7:16234. 10.1038/s41598-017-16563-3 29176590 PMC5701201

[B192] Zhang K. Tang X. Zhang J. Lu W. Lin X. Zhang Y. (2014). PEG-PLGA copolymers: their structure and structure-influenced drug delivery applications. *J. Control Release* 183 77–86. 10.1016/j.jconrel.2014.03.026 24675377

[B193] Zhang H. Xuan X. Yang S. Li X. Xu C. Gao X. (2018). Selection of viable human spermatozoa with low levels of DNA fragmentation from an immotile population using density gradient centrifugation and magnetic-activated cell sorting. *Andrologia* 50:e12821. 10.1111/and.12821 28466479

[B194] Zhang L. Gu F. X. Wang A. Z. Langer R. S. Farokhzad O. C. (2008). Nanoparticles in medicine: therapeutic applications and developments. *Clin. Pharmacol. Ther.* 83 761–769. 10.1038/sj.clp 17957183

[B195] Zhang Q. Kuang G. He S. Lu H. Cheng Y. Zhou D. (2020). Photoactivatable prodrug-backboned polymeric nanoparticles for efficient light-controlled gene delivery and synergistic treatment of platinum-resistant ovarian cancer. *Nano. Lett.* 20 3039–3049. 10.1021/acs.nanolett.9b04981 32250633

[B196] Zhang X. Chen J. Kang Y. Hong S. Zheng Y. Sun H. (2013). Targeted paclitaxel nanoparticles modified with follicle-stimulating hormone beta 81-95 peptide show effective antitumor activity against ovarian carcinoma. *Int. J. Pharm.* 453 498–505. 10.1016/j.ijpharm.2013.06.038 23811008

[B197] Zhang X. Y. Chen J. Zheng Y. F. Gao X. L. Kang Y. Liu J. C. (2009). Follicle-stimulating hormone peptide can facilitate paclitaxel nanoparticles to target ovarian carcinoma in vivo. *Cancer Res.* 69 6506–6514. 10.1158/0008-5472.CAN-08-4721 19638590

[B198] Zhang Y. Dong Y. Fu H. Huang H. Wu Z. Zhao M. (2020). Multifunctional tumor-targeted PLGA nanoparticles delivering Pt(IV)/siBIRC5 for US/MRI imaging and overcoming ovarian cancer resistance. *Biomaterials* 269:120478. 10.1016/j.biomaterials.2020.120478 33213862

[B199] Zhang Z. G. Buller B. Chopp M. (2019). Exosomes - beyond stem cells for restorative therapy in stroke and neurological injury. *Nat. Rev. Neurol.* 15 193–203. 10.1038/s41582-018-0126-4 30700824

[B200] Zhao M. D. Li J. Q. Chen F. Y. Dong W. Wen L. J. Fei W. D. (2019). Co-delivery of curcumin and paclitaxel by “core-shell” targeting amphiphilic copolymer to reverse resistance in the treatment of ovarian cancer. *Int J Nanomedicine* 14 9453–9467. 10.2147/IJN.S224579 31819443 PMC6898996

[B201] Zhou M. Nakatani E. Gronenberg L. S. Tokimoto T. Wirth M. J. Hruby V. J. (2007). Peptide-labeled quantum dots for imaging GPCRs in whole cells and as single molecules. *Bioconjug. Chem.* 18, 323–332. 10.1021/bc0601929 17373766

[B202] Zhu C. Chen Z. Gao S. Goh B. L. Samsudin I. B. Lwe K. W. (2019). Recent advances in non-toxic quantum dots and their biomedical applications. *Prog. Nat. Sci. Mater. Int.* 29, 628–640. 10.1016/j.pnsc.2019.11.007

[B203] Ziarati N. Tavalaee M. Bahadorani M. Nasr Esfahani M. H. (2017). Clinical outcomes of magnetic activated sperm sorting in infertile men candidate for ICSI. *Hum. Fertil.* 22, 118–125. 10.1080/14647273.2018.1424354 29363381

