# Supplementary material for: Biocompatible Nanomaterials as an Emerging Technology in Reproductive Health; a Focus on the Male
Source: Front Physiol. 2021 Nov 11;12:753686. doi: 10.3389/fphys.2021.753686 (PMC8632065; doi:10.3389/fphys.2021.753686)
Supplement: Supplementary file 1 [file Table_1.docx]

**Supplementary Table 1:** Recent research articles with reference to nanoparticle use in the male reproductive system. Abbreviations are defined at base of table.

| **Study Aim** | **Strategy** | **Outcome** | **author** |
| --- | --- | --- | --- |
| Develop targeted treatment of castration resistant prostate cancer | Used a polycationic NP -Folate-polyethylenimine 600-cyclodextrin (folate targeting via folate receptor) encapsulating a DNA damage repair inhibitor Dbait and docetaxel | Significantly improved the efficiency of radio-chemotherapy. *In vitro*- catrastration resistant prostate cancer cell lines, including androgen-receptor positive (PC-3 and DU145) and androgen receptor negative (LNCaP) cell lines. *In vivo* – PC-3 tumour model in nude mice. | Liu(Liu et al., 2020) |
| Testing the safety and efficacy of BIND-014 for use in targeted prostate cancer chemotherapy | BIND-014, a pharmaceutical product that is composed of docetaxel encapsulated in a PEGylated NP targeted with small molecule PSMA- targeting ligands. Phase 1 (52 patients) clinical trial(Von Hoff et al., 2016). and Phase 2 (42 patients with chemotherapy-naïve metastatic castration resistant prostate cancer(mCRPC)(Autio et al., 2018) | BIND-014 is active and well tolerated in patients with chemotherapy-naive mCRPC. The authors concluded that the observed antitumor activity may be related to PSMA expression levels on circulating tumour cells, which allows patients who are likely to benefit from this treatment to be identified before treatment is initiated. | Von Hoff et al. 2016(Von Hoff et al., 2016);  Autio et al., 2018(Autio et al., 2018) |
| Develop a dual drug therapy to treat prostate cancer | Delivery of two anticancer drugs, plumbagin and genistein, encapsulated in a L-α-phosphatidylcholine with 1,2-dipalmitoyl-sn-glycero-3-phosphoetahnolamine-N-methoxyPEG nanoparticle. Used *in vitro* PC-3 and LNCaP cells. *In vivo* IV injection into model of the same cells injected into female athymic nude mice. | The combination of plumbagin and genistein drugs was found inhibiting xenograft prostate tumor growth by ∼80 % with minimal toxicity. The authors attributed the observed anti-tumor effect to a decrease in proliferative cells and blood vessels. | Song et al. 2020(Song et al., 2020) |
| Dual imaging using fluorescence and magnetic resonance imaging (MRI) for early detection of prostate cancer | Near infrared fluorescent reagent, Cy7, and a contrast reagent, manganese oxide in a MSNP targeted with PSMA. Applied i*n vitro* and *in vivo* | Demonstrated that PSA targeted fluorescence and MR dual-functional nanoparticle can visualize prostate cancer and can be used as NIRF/MR contrast agents | Du et al. 2020(Du et al., 2020) |
| Concurrent treatment and MRI imaging for prostate cancer. To improve safety and efficacy of cabazitaxel (which is a new taxane-based drug and is currently delivered with Tween 80) and facilitate diagnosis | Gadolinium and cabazitaxel encapsulated in bovine serum albumin NPs. Applied *in vitro* to prostate cancer cell line and an *in vivo* prostate cancer rat model. | Showed enhanced uptake *in vitro* as well as improved retention time, antitumour inhibitory effect and safer profile than cabazitaxel-Tween 80 *in vivo*. This nanosystem can act as a therapeutic drug carrier to deliver cabazitaxel into prostate cancer and realize the integration of diagnosis and therapy. | Wan et al. 2020(Wan et al., 2020) |
| Concurrent targeted treatment and imaging of castration-resistant prostate cancer | Docetaxel and a cluster of SPIONs encapsulated in a PLGA-PEG NP targeted using Wy5a aptamers (targets castration resistant prostate cancer cell line, PC-3). Applied to PC-3 cells *in vitro* and *in vivo* xenograft mouse model | The targeted NPs showed a controlled drug release and an increased contrast-enhanced MRI capability. Wy5a improved cancer-targeted delivery to PC-3 cells *in vitro* and *in vivo*. *In vitro* studies demonstrated ultrasensitive MRI and increased cytotoxicity. *In vivo* NPs showed efficacious antitumor capability without significant systemic toxicity. | Fang et al. 2020(Fang et al., 2020) |
| Synergistic combination drug therapy for targeted prostate cancer treatment | Co-delivery of curcumin (CUR) and cabazitaxel (CTX) encapsulated in a PLGA-PEG NP targeted with the anti-PSMA aptamer A10-3.2 Applied to prostate cancer cells *in vitro* and tumour xenograft *in vivo.* | Both drugs had sustained released from the NPs. Aptamer-functionalized NPs exhibited good cell inhibition ability, high tumour accumulation, and tumour inhibition efficiency at the drug ratio of 2:5 (CUR:CTX). Demonstrated the potential of for prostate cancer. | Chen et al. 2020(Chen et al., 2020) |
| Development, characterization and assessment of *in vitro* efficacy of a natural product nanosystems for prostate cancer therapy | The natural product, Uncaria tomentose (UT) extracts, encapsulated in poly-e-caprolactone (PCL) or in PLGA NPs and applied to *in vitro* prostate cancer cell lines (LNCaP and DU145) | The NPs showed high drug loading. *In vitro*, both formulations reduced the viability of prostate cancer cells (DU145 and LNCaP cells). However, the UT-PLGA NPs showed higher cytotoxicity towards DU145 cells while the UT-PCL against LNCaP cells. | Ribeiro et al. 2020(Ribeiro et al., 2020) |
| Synergistic combination targeted drug therapy for prostate cancer treatment | Docetaxel and doxorubicin (DDC) packaged in hyaluronic acid (targets to CD44 protein) and cationic amphipathic starch NP. Applied *in vitro* PC-3 cells and *in vivo* PC-3 cell xenograft mouse model | *In vitro*, the DDC-NPs were as effective as the dual drug combination alone in terms of cytotoxicity, antimigration, and apoptosis. DDC-NPs targeted and fully released the payloads into cells. Low toxicity *in vivo* was confirmed and distribution *in vivo* showed that DDC- NPs enhanced tumour accumulation of drugs and decreased off-target localisation. Significant inhibition of xenograft tumour growth indicated that DDC acted synergistically after delivery by NPs. | Li et al. 2019(Li et al., 2019) |
| NP to deliver androgen deprivation therapy and chemotherapy concurrently to prostate cancer | Abiraterone acetate (CYP17a inhibitor) and doxetaxel packaged in a PLGA NP and delivered *in vitro* to a LNCaP cell line | NPs provided prolonged release of the drugs and maintenance of their concentration. NPs loaded with Abiraterone and doxetaxel exhibited a high cytotoxic activity on the LNCaP prostate cancer cell line, similar to the combination of free drugs and demonstrated a synergistic mechanism of action. | Sokol et al. 2019(Sokol et al., 2019) |
| Purification of porcine sperm to improve quality for use in artificial insemination | Lectin-coated magnetic IONPs to treat semen ejaculates prior to artificial insemination | Magnetic nanoparticles exhibited no toxic effects on sperm  fertilization capacity and piglet viability. Sperm had higher motility and viability of pregnancy was increased. Piglet birth weights and survival rates were higher. | Feugang et al. 2015(Feugang et al., 2015) |
| Purification of camel sperm to improve quality for use in artificial insemination | As preparation for artificial insemination used protease-based semen liquefaction and then purification using Lectins-coated (PNA and LCA) and Annexin V-coated magnetic IONPs | Protease-liquefication of semen followed by sperm magnetic nano-purification produced sperm that showed a dramatic improvement in post-thaw motility, viability, morphology, intact acrosome, HOST-reacted spermatozoa and reduction in sperm DNA fragmentation level. | Rateb 2020(Rateb, 2020) |
| Removal of abnormal sperm from boar semen intended for artificial insemination | Lectin-coated and Annexin V-coated magnetic IONPs to treat semen ejaculates prior to artificial insemination | Confirmed attachment of NPs to damaged spermatozoa. All sperm parameters and fertility showed no difference between the NP selected and the control sperm. However, the motility of nanoselected spermatozoa was improved. | Durfey et al. 2019(Durfey et al., 2019) |
| Protection of the reproduction system from genotoxicity from chemotherapeutics using nanosystems | Liposomes encapsulating doxorubicin (genotoxic chemotherapeutic agent) Researchers compared the mutagenicity of the free drug – doxorubicin hydrochloride with its nanoencapsulated form – doxorubicin loaded liposome, using conventional methods required for regulatory approval | Compared to the free doxorubicin, the doxorubicin-liposome expressed a significantly lower mutant frequency in the Ames assay, and was nongenotoxic in the *in vitro* micronucleus assay. However, release of the encapsulated drug prior to the Ames test show comparable mutagenic potential of the nanotherapeutic system to a free drug. | Alexander et al 2017(Alexander et al., 2017) |
| Non-surgical sterilisation of male animals – proof of concept *in vitro*. | Liposomal NP encapsulating α-mangostin (AM-NLC) for intratesticular injection. Proof of concept *in vitro* using spermatogonial cell line (GC-1) and cat testicular explants | AM-NLC exhibited antiproliferative activity towards and induced apoptosis in spermatogonium cells. AM-NLC exhibited anti-inflammatory activities in lipopolysaccharide-activated macrophages. Abnormal anatomy of seminiferous tubule was observed following treatment of testicular explant with AM-NLC. | Yostawonkul 2017(Yostawonkul et al., 2017) |
| Non-surgical sterilisation of male animals – proof of concept *in vitro*. | Doxorubicin packaged in liposomal (nanoemulsion) NP and applied to isolated and cultured rat seminiferous tubules (spermatogenic cells) | This nanosystem exhibited activation of the apoptosis pathway and inhibition of proliferation in spermatogenic cells. The nanosystem also precipitated anti-inflammatory activity in lipopolysaccharide stimulated macrophages. Treatment of isolated and cultured rat seminiferous tubules resulted in observable cell death. | Pagseesing 2018(Pagseesing et al., 2018) |
| Rescue of male infertility by restoring intratesticular peptidyl prolyl isomerase 1 (PIN1). | Used fibroin nanoparticle-encapsulated cationic lipid complex (Fibroplex) to deliver peptidyl prolyl isomerase 1 (PIN1) by intratesticular injection | Delivery of PIN1 via Fibroplex in Pin1 knockout testes produced fertile mice, achieving recovery from the infertile phenotypes. *In vivo* PIN1-loaded Fibroplex delivered intratesticularly to spermatogonial cells and Sertoli cells. The *in situ* sustained release of PIN1 restored gene expression, resulting in proliferation of spermatogonial cells and BTB integrity in Pin1 knockout testes. | Kim et al. 2020(Kim et al., 2020) |
| *In vitro* removal of tumour cells from neonate spermatogonial cells for transplantation in testes after cancer treatment | Cisplatin-loaded PLGA NPs targeted with FA | The PLGA NPs affected higher activation of apoptosis in the tumour cells than the free drug. There was no pathological tumour in testes after transplantation with treated co-cultured cells. | Shabani et al. 2018(Shabani et al., 2018) |
| The amelioration of the negative impacts of methylphenidate (Ritalin) on the testes | Curcumin-loaded superparamagnetic iron oxide NPs (SPIONs) administered to Ritalin-treated adult male rats | Curcumin nanoparticles significantly enhanced the level of the serum testosterone and improved measured parameters in sperm, round spermatids and Leydig cells in comparison with the Ritalin-only and vehicle control groups. Gene expression in inflammation pathways and apoptosis genes was significantly reduced in the NP treatment group in comparison with the control groups. | Raoofi et al. 2020 (Raoofi et al., 2020) |
| Method for removal of damaged sperm from bull semen samples  *in vitro* prior to assisted insemination | Used Cell-SELEX to develop ssDNA aptamers that selectively bind to damaged sperm cells. Attached the biotinylated aptamers to streptavidin-coated superparamagnetic iron oxide NPs (SPIONs) to target damaged spermatozoa | Authors applied this technique successfully to both unsorted and sex-sorted sperm suspensions. They observed improved semen quality by significantly increasing the percentage of healthy sperm cells without affecting the rate of blastocyst cleavage. | Farini et al. 2016(Farini et al., 2016) |
| Method for a nontoxic bioluminescent tagging of sperm for use in biotechnology applications. | Firefly luciferase-conjugated to magnetic IONPs. The presence of magnetic IONPs allows for manipulation, sorting, or tracking of cells using magnetic techniques. | Binding interactions between the synthesized nanoparticles and spermatozoon were confirmed. Bioluminescence imaging and UV–visible-NIR microscopy results showed light emission from sperm samples incubated with the NPs. | Vasquez et al. 2016(Vasquez et al., 2016) |

FA- Folate; HOST- hypoosmotic swelling test, LNCaP –androgen receptor negative prostate cancer cells; NP- nanoparticle; IONP – iron oxide nanoparticle; PC-3 and DU145 - androgen-receptor positive prostate cancer cells; MSNP- mesoporous silica nanoparticle; PLGA- poly(lactic-co-glycolic acid); PSMA- Prostate-specific membrane antigen; PEG-polyethylene glycol; SPION- superparamagnetic iron oxide nanoparticle.

**REFERENCES**

Alexander, J.F., Aguirre-Villarreal, D., and Godin, B. (2017). Liposomal encapsulation masks genotoxicity of a chemotherapeutic agent in regulatory toxicology assessments. *Nanomedicine* 13(3)**,** 829-833. doi: 10.1016/j.nano.2016.12.016.

Autio, K.A., Dreicer, R., Anderson, J., Garcia, J.A., Alva, A., Hart, L.L., et al. (2018). Safety and Efficacy of BIND-014, a Docetaxel Nanoparticle Targeting Prostate-Specific Membrane Antigen for Patients With Metastatic Castration-Resistant Prostate Cancer: A Phase 2 Clinical Trial. *JAMA Oncol* 4(10)**,** 1344-1351. doi: 10.1001/jamaoncol.2018.2168.

Chen, Y., Deng, Y., Zhu, C., and Xiang, C. (2020). Anti prostate cancer therapy: Aptamer-functionalized, curcumin and cabazitaxel co-delivered, tumor targeted lipid-polymer hybrid nanoparticles. *Biomed Pharmacother* 127**,** 110181. doi: 10.1016/j.biopha.2020.110181.

Du, D., Fu, H.J., Ren, W.W., Li, X.L., and Guo, L.H. (2020). PSA targeted dual-modality manganese oxide-mesoporous silica nanoparticles for prostate cancer imaging. *Biomed Pharmacother* 121**,** 109614. doi: 10.1016/j.biopha.2019.109614.

Durfey, C.L., Swistek, S.E., Liao, S.F., Crenshaw, M.A., Clemente, H.J., Thirumalai, R., et al. (2019). Nanotechnology-based approach for safer enrichment of semen with best spermatozoa. *J Anim Sci Biotechnol* 10**,** 14. doi: 10.1186/s40104-018-0307-4.

Fang, Y., Lin, S., Yang, F., Situ, J., Lin, S., and Luo, Y. (2020). Aptamer-Conjugated Multifunctional Polymeric Nanoparticles as Cancer-Targeted, MRI-Ultrasensitive Drug Delivery Systems for Treatment of Castration-Resistant Prostate Cancer. *Biomed Res Int* 2020**,** 9186583. doi: 10.1155/2020/9186583.

Farini, V.L., Camano, C.V., Ybarra, G., Viale, D.L., Vichera, G., Yakisich, J.S., et al. (2016). Improvement of bovine semen quality by removal of membrane-damaged sperm cells with DNA aptamers and magnetic nanoparticles. *J Biotechnol* 229**,** 33-41. doi: 10.1016/j.jbiotec.2016.05.008.

Feugang, J.M., Liao, S.F., Crenshaw, M.A., Clemente, H., Willard, S.T., and Ryan, P.L. (2015). Lectin-Functionalized Magnetic Iron Oxide Nanoparticles for Reproductive Improvement. *Journal of Fertilization: In Vitro - IVF-Worldwide, Reproductive Medicine, Genetics and Stem Cell* 03(02)**,** 145. doi: 10.4172/2375-4508.1000145.

Kim, W.J., Kim, B.S., Kim, H.J., Cho, Y.D., Shin, H.L., Yoon, H.I., et al. (2020). Intratesticular Peptidyl Prolyl Isomerase 1 Protein Delivery Using Cationic Lipid-Coated Fibroin Nanoparticle Complexes Rescues Male Infertility in Mice. *ACS Nano* 14(10)**,** 13217-13231. doi: 10.1021/acsnano.0c04936.

Li, K., Zhan, W., Chen, Y., Jha, R.K., and Chen, X. (2019). Docetaxel and Doxorubicin Codelivery by Nanocarriers for Synergistic Treatment of Prostate Cancer. *Front Pharmacol* 10**,** 1436. doi: 10.3389/fphar.2019.01436.

Liu, N., Ji, J., Qiu, H., Shao, Z., Wen, X., Chen, A., et al. (2020). Improving radio-chemotherapy efficacy of prostate cancer by co-deliverying docetaxel and dbait with biodegradable nanoparticles. *Artif Cells Nanomed Biotechnol* 48(1)**,** 305-314. doi: 10.1080/21691401.2019.1703726.

Pagseesing, S., Yostawonkul, J., Surassmo, S., Boonrungsiman, S., Namdee, K., Khongkow, M., et al. (2018). Formulation, physical, in vitro and ex vivo evaluation of nanomedicine-based chemosterilant for non-surgical castration of male animals. *Theriogenology* 108**,** 167-175. doi: 10.1016/j.theriogenology.2017.12.014.

Raoofi, A., Delbari, A., Mahdian, D., Mojadadi, M.S., Akhlaghi, M., Dadashizadeh, G., et al. (2020). Effects of curcumin nanoparticle on the histological changes and apoptotic factors expression in testis tissue after methylphenidate administration in rats. *Acta Histochem* 123(1)**,** 151656. doi: 10.1016/j.acthis.2020.151656.

Rateb, S.A. (2020). Purification of cryopreserved camel spermatozoa following protease-based semen liquefaction by lectin-functionalized DNA-defrag magnetic nanoparticles. *Reprod Domest Anim*. doi: 10.1111/rda.13863.

Ribeiro, A.F., Santos, J.F., Mattos, R.R., Barros, E.G.O., Nasciutti, L.E., Cabral, L.M., et al. (2020). Characterization and in vitro antitumor activity of polymeric nanoparticles loaded with Uncaria tomentosa extract. *An Acad Bras Cienc* 92(1)**,** e20190336. doi: 10.1590/0001-3765202020190336.

Shabani, R., Ashjari, M., Ashtari, K., Izadyar, F., Behnam, B., Khoei, S., et al. (2018). Elimination of mouse tumor cells from neonate spermatogonial cells utilizing cisplatin-entrapped folic acid-conjugated poly(lactic-co-glycolic acid) nanoparticles in vitro. *Int J Nanomedicine* 13**,** 2943-2954. doi: 10.2147/IJN.S155052.

Sokol, M.B., Nikolskaya, E.D., Yabbarov, N.G., Zenin, V.A., Faustova, M.R., Belov, A.V., et al. (2019). Development of novel PLGA nanoparticles with co-encapsulation of docetaxel and abiraterone acetate for a highly efficient delivery into tumor cells. *J Biomed Mater Res B Appl Biomater* 107(4)**,** 1150-1158. doi: 10.1002/jbm.b.34208.

Song, Y.Y., Yuan, Y., Shi, X., and Che, Y.Y. (2020). Improved drug delivery and anti-tumor efficacy of combinatorial liposomal formulation of genistein and plumbagin by targeting Glut1 and Akt3 proteins in mice bearing prostate tumor. *Colloids Surf B Biointerfaces* 190**,** 110966. doi: 10.1016/j.colsurfb.2020.110966.

Vasquez, E.S., Feugang, J.M., Willard, S.T., Ryan, P.L., and Walters, K.B. (2016). Bioluminescent magnetic nanoparticles as potential imaging agents for mammalian spermatozoa. *J Nanobiotechnology* 14**,** 20. doi: 10.1186/s12951-016-0168-y.

Von Hoff, D.D., Mita, M.M., Ramanathan, R.K., Weiss, G.J., Mita, A.C., LoRusso, P.M., et al. (2016). Phase I Study of PSMA-Targeted Docetaxel-Containing Nanoparticle BIND-014 in Patients with Advanced Solid Tumors. *Clin Cancer Res* 22(13)**,** 3157-3163. doi: 10.1158/1078-0432.CCR-15-2548.

Wan, Z., Xie, F., Wang, L., Zhang, G., and Zhang, H. (2020). Preparation and Evaluation of Cabazitaxel-Loaded Bovine Serum Albumin Nanoparticles for Prostate Cancer. *Int J Nanomedicine* 15**,** 5333-5344. doi: 10.2147/IJN.S258856.

Yostawonkul, J., Surassmo, S., Namdee, K., Khongkow, M., Boonthum, C., Pagseesing, S., et al. (2017). Nanocarrier-mediated delivery of alpha-mangostin for non-surgical castration of male animals. *Sci Rep* 7(1)**,** 16234. doi: 10.1038/s41598-017-16563-3.
